# Supplementary material for: A patient-oriented research approach to assessing patients’ and primary care physicians’ opinions on trauma-informed care
Source: PLoS One. 2021 Jul 9;16(7):e0254266. doi: 10.1371/journal.pone.0254266 (PMC8270182; doi:10.1371/journal.pone.0254266)
Supplement: S1 File — Patient and physician trauma-informed care survey. (DOCX) [file pone.0254266.s001.docx]

# Patient Trauma-Informed Care Survey

**For each of the following questions, please consider your experience with your current primary care physician (i.e., general physician and family physician) in Canada. If you do not have a regular physician, please consider your most recent visit to a primary care physician. Please answer how often you have received the following services from your primary care physician and how important these services are to you. You may have to scroll across to see all the options.**

|  | Frequency | | | | | | Importance | | | | |
| --- | --- | --- | --- | --- | --- | --- | --- | --- | --- | --- | --- |
|  | Never | Seldom | Occasionally | To a considerable degree | Almost always | Not important | | Slightly important | Moderately important | Important | Very important |
| Provide a physical space that is not perceived as threatening (e.g., adequate lighting, private area for you to complete screening questionnaires, adequate and safe distance between you and your healthcare provider). |  |  |  |  |  |  | |  |  |  |  |
| Make you feel welcome by being warm and friendly, and using a welcoming tone of voice. |  |  |  |  |  |  | |  |  |  |  |
| Find out what is most pressing for you. |  |  |  |  |  |  | |  |  |  |  |
| Clarify your understanding of your reasons for seeking services. |  |  |  |  |  |  | |  |  |  |  |
| Communicate openly (e.g., provide all relevant information, being transparent) and initiate communication. |  |  |  |  |  |  | |  |  |  |  |
| Listen to you (e.g., paying attention to what you are saying, not interrupting you) and make eye contact with you when interacting with you. |  |  |  |  |  |  | |  |  |  |  |
| Allow and encourage expression of feelings (even negative emotions) without judgment (e.g. not asserting their judgement on you). |  |  |  |  |  |  | |  |  |  |  |
| Make you feel emotionally safe by providing reassurance and validating your experiences. |  |  |  |  |  |  | |  |  |  |  |
| Use professional tone to convey genuine concern. |  |  |  |  |  |  | |  |  |  |  |
| Respond appropriately to your nonverbal communication. |  |  |  |  |  |  | |  |  |  |  |
| Ask about adverse (stressful) childhood experiences and past emotional trauma. |  |  |  |  |  |  | |  |  |  |  |
| Respond in a patient-focused manner if you were to disclose past trauma. |  |  |  |  |  |  | |  |  |  |  |
| Explain things in a way you could understand (e.g., use plain language without jargon). |  |  |  |  |  |  | |  |  |  |  |
| Provide informed consent prior to performing any medical activities (e.g., touching you or any medical examinations). |  |  |  |  |  |  | |  |  |  |  |
| Explain rationale before performing something. |  |  |  |  |  |  | |  |  |  |  |
| Check for your understanding of information provided |  |  |  |  |  |  | |  |  |  |  |
| Acknowledge and take responsibility for their miscommunications. |  |  |  |  |  |  | |  |  |  |  |
| Recognize and equalize power imbalances (e.g., understanding you are an expert in your own life, speaking to you at your level). |  |  |  |  |  |  | |  |  |  |  |
| Empowering you by providing encouragement, using positive language, and avoiding criticism. |  |  |  |  |  |  | |  |  |  |  |
| Use statements (e.g., “It’s your decision,” “It’s not me to decide”) that make collaboration and choice explicit. |  |  |  |  |  |  | |  |  |  |  |
| Outlining consent and format of treatment. |  |  |  |  |  |  | |  |  |  |  |
| Help you identify your strengths and skills that may facilitate management of your own care. |  |  |  |  |  |  | |  |  |  |  |
| Help you identify your challenges and difficulties that may get in the way of treatment. |  |  |  |  |  |  | |  |  |  |  |
| Provide you with choices that fit your life circumstances for treatment preferences. |  |  |  |  |  |  | |  |  |  |  |
| Involve you in decisions surrounding your care. |  |  |  |  |  |  | |  |  |  |  |
| Inquire about others who may be helpful to include in your care (e.g., family member). |  |  |  |  |  |  | |  |  |  |  |
| Awareness of your cultural identity and sexuality. |  |  |  |  |  |  | |  |  |  |  |
| Ask you about your cultural beliefs of your health and illness, and how you prefer to treat yourself. |  |  |  |  |  |  | |  |  |  |  |
| Respect your cultural preference for treatment. |  |  |  |  |  |  | |  |  |  |  |

# Trauma-Informed Care Survey—Physician Version

**It is recognized that physicians have busy schedules. While some behaviours are important, it is not always possible to carry them out in light of a busy caseload. Please do your best to accurately respond to the following questions. For each of the following questions, please answer how frequently you provide the following services to your patients and how important providing these services are to you. If you responded “Never,” “Seldom,” or “Occasionally” to the any of the items below, please select possible reasons for not performing these services more often. You may have to scroll across to see all the options.**

|  | **Frequency**  1 – Never  2 – Seldom  3 – Occasionally  4- To a considerable degree  5 – Almost always | | | | | **Importance**  1 – Not important  2 – Slightly important  3 – Moderately important  4 – Important  5 – Very important | | | | | **Reasons** | | | | | | |
| --- | --- | --- | --- | --- | --- | --- | --- | --- | --- | --- | --- | --- | --- | --- | --- | --- | --- |
|  | 0 | 1 | 2 | 3 | 4 | 0 | 1 | 2 | 3 | 4 | Not relevant to current problem | Lower priority | Lack of time | Lack of training | Feelings of discomfort | Lack of Resources | Not knowing available resources |
| Provide a physical space that is not perceived as threatening (e.g., adequate lighting, private area for your patients to complete screening questionnaires, adequate and safe distance between you your patients). |  |  |  |  |  |  |  |  |  |  |  |  |  |  |  |  |  |
| Make your patients feel welcome by being warm and friendly, and using a welcoming tone of voice. |  |  |  |  |  |  |  |  |  |  |  |  |  |  |  |  |  |
| Find out what is most pressing for your patients. |  |  |  |  |  |  |  |  |  |  |  |  |  |  |  |  |  |
| Clarify your patients' understanding of their reasons for seeking services. |  |  |  |  |  |  |  |  |  |  |  |  |  |  |  |  |  |
| Communicate openly (e.g., provide all relevant information, being transparent) and initiate communication. |  |  |  |  |  |  |  |  |  |  |  |  |  |  |  |  |  |
| Listen to your patients (e.g., paying attention to what they are saying, not interrupting them) and make eye contact with your patients when interacting with them. |  |  |  |  |  |  |  |  |  |  |  |  |  |  |  |  |  |
| Allow and encourage expression of feelings (even negative emotions) without judgment (e.g. not asserting your judgement on your patients). |  |  |  |  |  |  |  |  |  |  |  |  |  |  |  |  |  |
| Make your patients feel emotionally safe by providing reassurance and validating their experiences. |  |  |  |  |  |  |  |  |  |  |  |  |  |  |  |  |  |
| Use professional tone to convey genuine concern. |  |  |  |  |  |  |  |  |  |  |  |  |  |  |  |  |  |
| Respond appropriately to your patients' nonverbal communication. |  |  |  |  |  |  |  |  |  |  |  |  |  |  |  |  |  |
| Ask about adverse (stressful) childhood experiences and past emotional trauma. |  |  |  |  |  |  |  |  |  |  |  |  |  |  |  |  |  |
| Respond in a patient-focused manner when your patients disclose past trauma. |  |  |  |  |  |  |  |  |  |  |  |  |  |  |  |  |  |
| Explain things in a way your patients could understand (e.g., use plain language without jargon). |  |  |  |  |  |  |  |  |  |  |  |  |  |  |  |  |  |
| Provide informed consent prior to performing any medical activities (e.g., touching your patients or any medical examinations). |  |  |  |  |  |  |  |  |  |  |  |  |  |  |  |  |  |
| Explain rationale before performing something. |  |  |  |  |  |  |  |  |  |  |  |  |  |  |  |  |  |
| Check for your patients' understanding of information provided |  |  |  |  |  |  |  |  |  |  |  |  |  |  |  |  |  |
| Acknowledge and take responsibility for your miscommunications. |  |  |  |  |  |  |  |  |  |  |  |  |  |  |  |  |  |
| Recognize and equalize power imbalances (e.g., understanding your patients are an expert in their own life, speaking to your patients at their level). |  |  |  |  |  |  |  |  |  |  |  |  |  |  |  |  |  |
| Empower your patients by providing encouragement, using positive language, and avoiding criticism. |  |  |  |  |  |  |  |  |  |  |  |  |  |  |  |  |  |
| Use statements (e.g., “It’s your decision,” “It’s not me to decide”) that make collaboration and choice explicit |  |  |  |  |  |  |  |  |  |  |  |  |  |  |  |  |  |
| Outline consent and format of treatment. |  |  |  |  |  |  |  |  |  |  |  |  |  |  |  |  |  |
| Help your patients identify their strengths and skills that may facilitate management of their own care. |  |  |  |  |  |  |  |  |  |  |  |  |  |  |  |  |  |
| Help your patients identify their challenges and difficulties that may get in the way of treatment. |  |  |  |  |  |  |  |  |  |  |  |  |  |  |  |  |  |
| Provide your patients with choices and choices that fit their life circumstances for treatment preferences. |  |  |  |  |  |  |  |  |  |  |  |  |  |  |  |  |  |
| Involving your patients in decisions surrounding their care. |  |  |  |  |  |  |  |  |  |  |  |  |  |  |  |  |  |
| Inquire about others who may be helpful to include in your patients' care (e.g., family member). |  |  |  |  |  |  |  |  |  |  |  |  |  |  |  |  |  |
| Awareness of your patients' cultural identity and sexuality. |  |  |  |  |  |  |  |  |  |  |  |  |  |  |  |  |  |
| Ask your patients about your cultural beliefs of their health and illness, and how they prefer to treat themselves. |  |  |  |  |  |  |  |  |  |  |  |  |  |  |  |  |  |
| Respect your patients' cultural preference for treatment. |  |  |  |  |  |  |  |  |  |  |  |  |  |  |  |  |  |
