# Supplementary material for: A patient-oriented research approach to assessing patients’ and primary care physicians’ opinions on trauma-informed care
Source: PLoS One. 2021 Jul 9;16(7):e0254266. doi: 10.1371/journal.pone.0254266 (PMC8270182; doi:10.1371/journal.pone.0254266)
Supplement: S2 File — Patient and physician survey for Phase 2. (DOCX) [file pone.0254266.s002.docx]

# Phase 2 Recommendations—Patient Version

*A concern brought up by some of our patient participants was that the appointment duration was too short. The billing system for appointments was a constraint brought up by our physician advisor as physicians may be limited in their capacity to book for longer appointment times.*

| If there was a mechanism in place for booking longer appointment times ahead of time, how helpful would it be to you?   - Extremely helpful   Very helpful  Moderately helpful  Slightly helpful  Not at all helpful | How likely would you utilize this option?   - Very likely   Likely  Neither likely nor unlikely  Unlikely  Very unlikely |
| --- | --- |

| If there was a mechanism in place for your physician where they were able to extend your appointment time should a situation come up unexpectedly, how helpful would it be to you?  Extremely helpful  Very helpful  Moderately helpful  Slightly helpful  Not at all helpful | How likely would you utilize this option?  Very likely  Likely  Neither likely nor unlikely  Unlikely  Very unlikely |
| --- | --- |

How helpful would it be if your physician asked you complete an annual life stress inventory that will take 2-3 minutes to complete? (Examples of life stress: divorce or separation, marriage, being fired from work, changes in residence).

Extremely helpful

Very helpful

Moderately helpful

Slightly helpful

Not at all helpful

If your physician asked, how likely would you disclose this information?

Very likely

Likely

Neither likely nor unlikely

Unlikely

Very unlikely

*According to the American Psychiatric Association, events that can lead to traumatic stress include “exposure to actual or threatened death, serious injury or sexual violence.” These events may be directly experienced by the individual, witnessing the events happen to another individual, learning that these events happened to a close family member or friend, and/or indirect exposure to details of the events (such as in the case of first responders). Adverse childhood experiences may include abuse include abuse (i.e., emotional, physical, and sexual abuse), household challenges (i.e., mother treated violently, household substance abuse, mental illness in household, parental separation or divorce, and criminal household members), and neglect (i.e., emotional and physical neglect).*

How helpful would it be if your physician asked you complete a brief trauma screening questionnaire to inquire about your past traumatic events and adverse childhood experiences that will take 2-3 minutes to complete?

Extremely helpful

Very helpful

Moderately helpful

Slightly helpful

Not at all helpful

If your physician asked, how likely would you disclose this information?

Very likely

Likely

Neither likely nor unlikely

Unlikely

Very unlikely

If you have ever experienced past trauma or have a family member who experienced past trauma, how helpful would it be to have the following kinds of information pamphlets about coping with trauma? How likely would you refer to each of these pamphlets for information?

| **Kinds of information pamphlet** | **Helpfulness**  5 – Extremely helpful  4 – Very helpful  3 – Moderately helpful  2 – slightly helpful  1 – Not at all helpful | **Likelihood of referring to the pamphlet**  5 – Very likely  4 – Likely  3 – Neither likely nor unlikely  2 – Likely  1 – Very unlikely |
| --- | --- | --- |
| Information on different kinds trauma and how to cope with trauma |  |  |
| How trauma impacts physical and mental health |  |  |
| Understanding posttraumatic stress disorder (PTSD) and PTSD treatment |  |  |
| How trauma affect relationships |  |  |
| Disasters and traumatic loss |  |  |
| Traumatic stress and substance abuse problems |  |  |
| Intimate partner violence |  |  |
| Trauma as a result of medical errors |  |  |
| When a friend or loved one has been traumatized |  |  |
| Trauma information for parents |  |  |

| How helpful would it be to have a trauma resource centre **hotline** in your health region where patients can call to be provided with a list of resources?  Extremely helpful  Very helpful  Moderately helpful  Slightly helpful  Not at all helpful | How likely would you call in?  Very likely  Likely  Neither likely nor unlikely  Unlikely  Very unlikely |
| --- | --- |

| How helpful would it be to have a trauma resource centre **website** for your health region where patients can visit to access a list of resources?  Extremely helpful  Very helpful  Moderately helpful  Slightly helpful  Not at all helpful | How likely would you visit the website?  Very likely  Likely  Neither likely nor unlikely  Unlikely  Very unlikely |
| --- | --- |

For each of the following peer support groups for trauma survivors, would you find this kind of group beneficial for you? How likely would you use this resource?

| **Kinds of peer support groups** | **Helpfulness**  5 – Extremely helpful  4 – Very helpful  3 – Moderately helpful  2 – slightly helpful  1 – Not at all helpful | **Likelihood of joining in person group**  5 – Very likely  4 – Likely  3 – Neither likely nor unlikely  2 – Likely  1 – Very unlikely | **Likelihood of joining online group**  5 – Very likely  4 – Likely  3 – Neither likely nor unlikely  2 – Likely  1 – Very unlikely |
| --- | --- | --- | --- |
| Trauma support group for specific concerns (e.g., suicide, childhood abuse) |  |  |  |
| Trauma support group for general concerns |  |  |  |
| Navigating the healthcare system as a trauma survivor |  |  |  |
| Parent support group |  |  |  |

*A clinical pathway is a guideline of usual care for patients who have a certain disorder, implementing standards of care. Clinical pathways are also communication tools for patients and healthcare providers to ensure that patients receive appropriate and timely care. A trauma clinical pathway would include assessment of trauma in primary care and guidelines on the kind of professionals and resources to refer patients to. It could improve patient experience and outcomes for trauma survivors, and physicians would have guidelines for care.*

| If such a pathway was developed and implemented in your health region, how beneficial would it be to you?  Extremely helpful  Very helpful  Moderately helpful  Slightly helpful  Not at all helpful | How likely would you refer to this pathway to help guide your own patient experience?  Very likely  Likely  Neither likely nor unlikely  Unlikely  Very unlikely |
| --- | --- |

*Patient and family advisors on our research team said they appreciate it when physicians validate their emotions and experiences. A suggestion was physician training on how to manage and validate patient emotions, how to actively listen to patients and how to communicate effectively.*

| If such a training was available for physicians, how helpful do you think it would be for your family physician to receive this training?  Extremely helpful  Very helpful  Moderately helpful  Slightly helpful  Not at all helpful | What is the likelihood that it could positively impact your care if your family physician were to receive this training?  Very likely  Likely  Neither likely nor unlikely  Unlikely  Very unlikely |
| --- | --- |

*Sometimes it can be difficult to respond to and manage patients’ emotions. Research has shown that lack of self-care can lead to emotional exhaustion and apathy, while self-compassion and managing work-related stress may prevent this. Self-compassion training for physicians has been suggested by our patient, family and physician advisors. This training would include how to practice kindness and understanding towards oneself, and how to be mindful (i.e., being in the moment with one’s feelings and thoughts and being non-judgmental towards oneself). This could in turn help physicians to be kind and understanding towards others as well.*

| If self-compassion training was made available for physicians, do you think it would be helpful for family physicians to  receive this training?  Extremely helpful  Very helpful  Moderately helpful  Slightly helpful  Not at all helpful | How likely do you think this could positively impact your care if your family physician were to receive this training?  Very likely  Likely  Neither likely nor unlikely  Unlikely  Very unlikely |
| --- | --- |

*According to a Canadian study, the prevalence of trauma exposure is approximately 76%. In our study, 72% of patient participants reported they experienced at least one traumatic event in their lifetime. Trauma can lead to changes within individuals and these changes can impact health and behaviour. Sometimes trauma survivors may experience reduced trust in others and may not feel safe in certain situations. Trauma-informed care is a patient care approach that revolves around the following key principles:*

- *Understanding trauma and its long-term and pervasive impact on survivors*

*Promoting and providing a safe and welcoming environment*

*Building trust and being transparent*

*Peer support for patients*

*Developing collaborative relationships with patients*

*Provide strengths-based approach to empower and support patients*

*Understanding and recognizing that healing takes place within cultural contexts*

How important is it for your family physician to receive training in each of the principles?

| **Principles** | Extremely helpful | Very helpful | Moderately helpful | Slightly helpful | Not helpful at all |
| --- | --- | --- | --- | --- | --- |
| Understanding trauma and its long-term and pervasive impact on survivors |  |  |  |  |  |
| Promoting and providing a safe and welcoming environment |  |  |  |  |  |
| Building trust and being transparent |  |  |  |  |  |
| Peer support for patients |  |  |  |  |  |
| Developing collaborative relationships with patients |  |  |  |  |  |
| Provide strengths-based approach to empower and support patients |  |  |  |  |  |
| Understanding and recognizing that healing takes place within cultural contexts |  |  |  |  |  |

| If training for trauma-informed care was made available for physicians, do you think it would be helpful for family physicians to receive this training?  Extremely helpful  Very helpful  Moderately helpful  Slightly helpful  Not at all helpful | How likely of a positive impact do you think it will have to your care if your family physician were to receive this training?  Very likely  Likely  Neither likely nor unlikely  Unlikely  Very unlikely |
| --- | --- |

*Research has shown that women and individuals who are gender non-binary face gender-based discrimination and/or oppression. Our study showed that female participants placed trauma-informed care at higher importance than male participants. Examples of gender differences in trauma experiences include:*

- *Women are at a higher risk for intimate partner violence than men*

*Girls are more likely than boys to be sexually abused*

*Boys have a higher risk than girls to be physically abused*

| If training on gender issues related to trauma and healthcare was made available for physicians, do you think it would be helpful for family physicians to receive this training?  Extremely helpful  Very helpful  Moderately helpful  Slightly helpful  Not at all helpful | How likely do you think this could positively impact your care if your family physician were to receive this training?  Very likely  Likely  Neither likely nor unlikely  Unlikely  Very unlikely |
| --- | --- |

*Research shown different ethnic groups experience different types of traumatic events and varying proportions. Indigenous communities have endured various kinds of trauma and horrendous events. Some groups of newcomers to Canada faced traumatic events in their home countries and they may be marginalized in Canada. White participants in our study indicated that they received trauma-informed care at higher frequency than participants from other ethnic groups.*

| If training on ethnic issues surrounding trauma and healthcare was made available for physicians, do you think it would be helpful for family physicians to receive this training?  Extremely helpful  Very helpful  Moderately helpful  Slightly helpful  Not at all helpful | How likely do you think this could positively impact your care if your family physician were to receive this training?  Very likely  Likely  Neither likely nor unlikely  Unlikely  Very unlikely |
| --- | --- |

*There is research evidence that individuals belonging to marginalized groups (due to factors such as sexual orientation, religion, disability, or socioeconomic status) face discrimination and/or oppression. Discrimination and oppression, along with any past trauma, could have compounding effects on individuals’ mental and physical health.*

| If training on trauma and healthcare in marginalized groups was made available for physicians, do you think it would be helpful for family physicians to receive this training?  Extremely helpful  Very helpful  Moderately helpful  Slightly helpful  Not at all helpful | How likely do you think this could positively impact your care if your family physician were to receive this training?  Very likely  Likely  Neither likely nor unlikely  Unlikely  Very unlikely |
| --- | --- |

# Phase 2 Recommendations—Physician Version

*A concern brought up by some of our patient participants was that the appointment duration was too short. The billing system for appointments was a constraint brought up by our physician advisor as physicians may be limited in their capacity to book for longer appointment times.*

| If there was a mechanism in place for booking and billing longer appointment times ahead of time, how helpful would it be to you?  Extremely helpful  Very helpful  Moderately helpful  Slightly helpful  Not at all helpful | How likely would you utilize this option?  Very likely  Likely  Neither likely nor unlikely  Unlikely  Very unlikely |
| --- | --- |

| If there was a mechanism in place to bill for unexpected appointments that require longer visits, how helpful would it be to you?  Extremely helpful  Very helpful  Moderately helpful  Slightly helpful  Not at all helpful | How likely would you utilize this option?  Very likely  Likely  Neither likely nor unlikely  Unlikely  Very unlikely |
| --- | --- |

How helpful would it be to ask your patients to complete an annual life stress inventory that will take 2-3 minutes to complete? (Examples of life stress: divorce or separation, marriage, being fired from work, changes in residence).

Extremely helpful

Very helpful

Moderately helpful

Slightly helpful

Not at all helpful

How likely would you ask your patients?

Very likely

Likely

Neither likely nor unlikely

Unlikely

Very unlikely

*According to the American Psychiatric Association, events that can lead to traumatic stress include “exposure to actual or threatened death, serious injury or sexual violence.” These events may be directly experienced by the individual, witnessing the events happen to another individual, learning that these events happened to a close family member or friend, and/or indirect exposure to details of the events (such as in the case of first responders). Adverse childhood experiences may include abuse include abuse (i.e., emotional, physical, and sexual abuse), household challenges (i.e., mother treated violently, household substance abuse, mental illness in household, parental separation or divorce, and criminal household members), and neglect (i.e., emotional and physical neglect).*

How helpful would it be ask your patients to complete a brief trauma screening questionnaire to inquire about their past traumatic events and adverse childhood experiences that will take 2-3 minutes to complete?

Extremely helpful

Very helpful

Moderately helpful

Slightly helpful

Not at all helpful

How likely would you ask your patients?

Very likely

Likely

Neither likely nor unlikely

Unlikely

Very unlikely

How helpful would it be to have the following kinds of information pamphlets about coping with trauma to give to your patients? How likely would you provide each of these pamphlets to your patients who experienced traumatic events?

| **Kinds of information pamphlet** | **Helpfulness**  5 – Extremely helpful  4 – Very helpful  3 – Moderately helpful  2 – slightly helpful  1 – Not at all helpful | **Likelihood of referring your patients to the pamphlet**  5 – Very likely  4 – Likely  3 – Neither likely nor unlikely  2 – Likely  1 – Very unlikely |
| --- | --- | --- |
| Information on different kinds trauma and how to cope with trauma |  |  |
| How trauma impacts physical and mental health |  |  |
| Understanding posttraumatic stress disorder (PTSD) and PTSD treatment |  |  |
| How trauma affect relationships |  |  |
| Disasters and traumatic loss |  |  |
| Traumatic stress and substance abuse problems |  |  |
| Intimate partner violence |  |  |
| Trauma as a result of medical errors |  |  |
| When a friend or loved one has been traumatized |  |  |
| Trauma information for parents |  |  |

| How helpful would it be to have a trauma resource centre **hotline** in your health region where patients can call to be provided with a list of resources?  Extremely helpful  Very helpful  Moderately helpful  Slightly helpful  Not at all helpful | How likely would you refer your patients to this hotline?  Very likely  Likely  Neither likely nor unlikely  Unlikely  Very unlikely |
| --- | --- |

| How helpful would it be to have a trauma resource centre **website** for your health region where patients can visit to access a list of resources?  Extremely helpful  Very helpful  Moderately helpful  Slightly helpful  Not at all helpful | How likely would refer your patients to this website?  Very likely  Likely  Neither likely nor unlikely  Unlikely  Very unlikely |
| --- | --- |

For each of the following peer support groups for trauma survivors, would you find this kind of group beneficial for your patients?

How likely would you refer your patients to this resource?

| **Kinds of peer support groups** | **Helpfulness**  5 – Extremely helpful  4 – Very helpful  3 – Moderately helpful  2 – slightly helpful  1 – Not at all helpful | **Likelihood of referring your patients to in person group**  5 – Very likely  4 – Likely  3 – Neither likely nor unlikely  2 – Likely  1 – Very unlikely | **Likelihood of referring your patients to online group**  5 – Very likely  4 – Likely  3 – Neither likely nor unlikely  2 – Likely  1 – Very unlikely |
| --- | --- | --- | --- |
| Trauma support group for specific concerns (e.g., suicide, childhood abuse) |  |  |  |
| Trauma support group for general concerns |  |  |  |
| Navigating the healthcare system as a trauma survivor |  |  |  |
| Parent support group |  |  |  |

*A clinical pathway is a guideline of usual care for patients who have a certain disorder, implementing standards of care. Clinical pathways are also communication tools for patients and healthcare providers to ensure that patients receive appropriate and timely care. A trauma clinical pathway would include assessment of trauma in primary care and guidelines on the kind of professionals and resources to refer patients to. It could improve patient experience and outcomes for trauma survivors, and physicians would have guidelines for care.*

| If such a pathway was developed and implemented in your health region, how beneficial would it be for your practice?  Extremely helpful  Very helpful  Moderately helpful  Slightly helpful  Not at all helpful | How likely would you refer to this pathway to guide treatment for your patients?  Very likely  Likely  Neither likely nor unlikely  Unlikely  Very unlikely |
| --- | --- |

*Patient and family advisors on our research team said they appreciate it when physicians validate their emotions and experiences. A suggestion was physician training on how to manage and validate patient emotions, how to actively listen to patients and how to communicate effectively.*

| If such a training was available for physicians, how helpful would it be for you to receive this training?  Extremely helpful  Very helpful  Moderately helpful  Slightly helpful  Not at all helpful | How likely do you think it would have a positive impact on your practice?  Very likely  Likely  Neither likely nor unlikely  Unlikely  Very unlikely |
| --- | --- |

Please indicate how likely you would attend this training for each of the following format.

|  | Very likely | Likely | Neither likely nor unlikely | Unlikely | Very unlikely |
| --- | --- | --- | --- | --- | --- |
| Online |  |  |  |  |  |
| In person |  |  |  |  |  |
| CME credit offered |  |  |  |  |  |
| CME credit not offered |  |  |  |  |  |

If this training could be used towards your continuing medical education, how many hours would be ideal? _____

*Sometimes it can be difficult to respond to and manage patients’ emotions. Research has shown that lack of self-care can lead to emotional exhaustion and apathy, while self-compassion and managing work-related stress may prevent this. Self-compassion training for physicians has been suggested by our patient, family and physician advisors. This training would include how to practice kindness and understanding towards oneself, and how to be mindful (i.e., being in the moment with one’s feelings and thoughts and being non-judgmental towards oneself). This could in turn help physicians to be kind and understanding towards others as well.*

| If self-compassion training was made available for physicians, do you think it would be helpful for family physicians to receive this training?  Extremely helpful  Very helpful  Moderately helpful  Slightly helpful  Not at all helpful | How likely do you think it would have a positive impact on your practice?  Very likely  Likely  Neither likely nor unlikely  Unlikely  Very unlikely |
| --- | --- |

Please indicate how likely you would attend this training for each of the following format.

|  | Very likely | Likely | Neither likely nor unlikely | Unlikely | Very unlikely |
| --- | --- | --- | --- | --- | --- |
| Online |  |  |  |  |  |
| In person |  |  |  |  |  |
| CME credit offered |  |  |  |  |  |
| CME credit not offered |  |  |  |  |  |

If this training could be used towards your continuing medical education, how many hours would be ideal? _____

*According to a Canadian study, the prevalence of trauma exposure is approximately 76%. In our study, 72% of patient participants reported they experienced at least one traumatic event in their lifetime. Trauma can lead to changes within individuals and these changes can impact health and behaviour. Sometimes trauma survivors may experience reduced trust in others and may not feel safe in certain situations. Trauma-informed care is a patient care approach that revolves around the following key principles:*

Understanding trauma and its long-term and pervasive impact on survivors

Promoting and providing a safe and welcoming environment

Building trust and being transparent

Peer support for patients

Developing collaborative relationships with patients

Provide strengths-based approach to empower and support patients

Understanding and recognizing that healing takes place within cultural contexts

How important is it for you to receive training in each of the principles?

| **Principles** | Extremely helpful | Very helpful | Moderately helpful | Slightly helpful | Not helpful at all |
| --- | --- | --- | --- | --- | --- |
| Understanding trauma and its long-term and pervasive impact on survivors |  |  |  |  |  |
| Promoting and providing a safe and welcoming environment |  |  |  |  |  |
| Building trust and being transparent |  |  |  |  |  |
| Peer support for patients |  |  |  |  |  |
| Developing collaborative relationships with patients |  |  |  |  |  |
| Provide strengths-based approach to empower and support patients |  |  |  |  |  |
| Understanding and recognizing that healing takes place within cultural contexts |  |  |  |  |  |

| If training for trauma-informed care was made available for physicians, do you think it would be helpful for family physicians to receive this training?  Extremely helpful  Very helpful  Moderately helpful  Slightly helpful  Not at all helpful | How likely do you think it would have a positive impact on your practice?  Very likely  Likely  Neither likely nor unlikely  Unlikely  Very unlikely |
| --- | --- |

Please indicate how likely you would attend this training for each of the following format.

|  | Very likely | Likely | Neither likely nor unlikely | Unlikely | Very unlikely |
| --- | --- | --- | --- | --- | --- |
| Online |  |  |  |  |  |
| In person |  |  |  |  |  |
| CME credit offered |  |  |  |  |  |
| CME credit not offered |  |  |  |  |  |

If this training could be used towards your continuing medical education, how many hours would be ideal? _____

*Research has shown that women and individuals who are gender non-binary face gender-based discrimination and/or oppression. Our study showed that female participants placed trauma-informed care at higher importance than male participants. Examples of gender differences in trauma experiences include:*

*Women are at a higher risk for intimate partner violence than men*

*Girls are more likely than boys to be sexually abused*

*Boys have a higher risk than girls to be physically abused*

| If training on gender issues related to trauma and healthcare was made available for physicians, do you think it would be helpful for family physicians to receive this training?  Extremely helpful  Very helpful  Moderately helpful  Slightly helpful  Not at all helpful | How likely do you think it would have a positive impact on your practice?  Very likely  Likely  Neither likely nor unlikely  Unlikely  Very unlikely |
| --- | --- |

Please indicate how likely you would attend this training for each of the following format.

|  | Very likely | Likely | Neither likely nor unlikely | Unlikely | Very unlikely |
| --- | --- | --- | --- | --- | --- |
| Online |  |  |  |  |  |
| In person |  |  |  |  |  |
| CME credit offered |  |  |  |  |  |
| CME credit not offered |  |  |  |  |  |

If this training could be used towards your continuing medical education, how many hours would be ideal? _____

*Research shown different ethnic groups experience different types of traumatic events and varying proportions. Indigenous communities have endured various kinds of trauma and horrendous events. Some groups of newcomers to Canada faced traumatic events in their home countries and they may be marginalized in Canada. White participants in our study indicated that they received trauma-informed care at higher frequency than participants from other ethnic groups.*

| If training on ethnic issues surrounding trauma and healthcare was made available for physicians, do you think it would be helpful for family physicians to receive this training?  Extremely helpful  Very helpful  Moderately helpful  Slightly helpful  Not at all helpful | How likely do you think it would have a positive impact on your practice?  Very likely  Likely  Neither likely nor unlikely  Unlikely  Very unlikely |
| --- | --- |

Please indicate how likely you would attend this training for each of the following format.

|  | Very likely | Likely | Neither likely nor unlikely | Unlikely | Very unlikely |
| --- | --- | --- | --- | --- | --- |
| Online |  |  |  |  |  |
| In person |  |  |  |  |  |
| CME credit offered |  |  |  |  |  |
| CME credit not offered |  |  |  |  |  |

If this training could be used towards your continuing medical education, how many hours would be ideal? _____

*There is research evidenced that individuals belonging to marginalized groups (due to factors such as sexual orientation, religion, disability, or socioeconomic status) face discrimination and/or oppression. Discrimination and oppression, along with any past trauma, could have compounding effects on individuals’ mental and physical health.*

| If training on trauma and healthcare in marginalized groups was made available for physicians, do you think it would be helpful for family physicians to receive this training?  Extremely helpful  Very helpful  Moderately helpful  Slightly helpful  Not at all helpful | How likely do you think it would have a positive impact on your practice?  Very likely  Likely  Neither likely nor unlikely  Unlikely  Very unlikely |
| --- | --- |

Please indicate how likely you would attend this training for each of the following format.

|  | Very likely | Likely | Neither likely nor unlikely | Unlikely | Very unlikely |
| --- | --- | --- | --- | --- | --- |
| Online |  |  |  |  |  |
| In person |  |  |  |  |  |
| CME credit offered |  |  |  |  |  |
| CME credit not offered |  |  |  |  |  |

If this training could be used towards your continuing medical education, how many hours would be ideal? _____
