# Supplementary material for: A patient-oriented research approach to assessing patients’ and primary care physicians’ opinions on trauma-informed care
Source: PLoS One. 2021 Jul 9;16(7):e0254266. doi: 10.1371/journal.pone.0254266 (PMC8270182; doi:10.1371/journal.pone.0254266)
Supplement: Attachment — Submitted filename: PONE-D-20-27620_reviewer.pdf [file pone.0254266.s003.pdf]

PLOS ONE

A Patient Oriented Research Approach to Assessing Patients’ and Primary Care Physicians’ Opinions on Trauma-Informed Care

--Manuscript Draft--

**Manuscript Number:**	PONE-D-20-27620
**Article Type:**	Research Article
**Full Title:**	A Patient Oriented Research Approach to Assessing Patients’ and Primary Care Physicians’ Opinions on Trauma-Informed Care
**Short Title:**	Patients’ and Primary Care Physicians’ Opinions on Trauma-Informed Care
**Corresponding Author:**	Seint Kokokyi, PhD<br>University of Manitoba College of Medicine<br>Winnipeg, CANADA
**Keywords:**	Trauma informed care; patient oriented research; physician training; patient education; primary care; patient care
**Abstract:**	Objective To gather patients’ and primary care physicians’ (PCP) opinions on Trauma-Informed Care (TIC) and to investigate the acceptability of recommendations developed by patient, family, and physician advisors.<br>Design Cross-sectional research survey design and patient engagement.<br>Setting Canada, 2017 to 2019.<br>Participants English-speaking adults and licensed PCPs residing in Canada.<br>Main outcome measures Participants were given a battery of questionnaires including a list of physician actions and a list of recommendations consistent with TIC.<br>Results Patients and PCPs viewed TIC as important. Both patients and PCPs rated the following recommendations as helpful and likely to positively impact patient care: physician training, online trauma resource centres, information pamphlets, the ability to extend appointment times, and trauma clinical pathways. PCPs’ responses were significantly more positive than patients’ responses.<br>Conclusion TIC is important to patients and PCPs. Patients and PCPs believe changes to physician training, patient engagement, and systemic factors would be helpful and likely to positively impact patient care. Future research needs to be conducted to investigate whether these recommendations improve patient care.
**Order of Authors:**	Seint Kokokyi, PhD
	Bridget Klest
	Hannah Anstey
**Additional Information:**	
**Question**	**Response**
**Financial Disclosure**<br><br>Enter a financial disclosure statement that describes the sources of funding for the work included in this submission. Review the submission guidelines for detailed requirements. View published research articles from *PLOS ONE* for specific examples.<br><br>This statement is required for submission and **will appear in the published article** if the submission is accepted. Please make sure it is accurate.	B.K.<br>3134<br>Saskatchewan Health Research Foundation<br>https://www.shrf.ca/<br>The funders had no role in study design, data collection and analysis, decision to publish, or preparation of the manuscript.

Powered by Editorial Manager® and ProduXion Manager® from Aries Systems Corporation

**Unfunded studies**<br>Enter: *The author(s) received no specific funding for this work.*<br><br>**Funded studies**<br>Enter a statement with the following details:<br>• Initials of the authors who received each award<br>• Grant numbers awarded to each author<br>• The full name of each funder<br>• URL of each funder website<br>• Did the sponsors or funders play any role in the study design, data collection and analysis, decision to publish, or preparation of the manuscript?<br>• **NO** - Include this sentence at the end of your statement: *The funders had no role in study design, data collection and analysis, decision to publish, or preparation of the manuscript.*<br>• **YES** - Specify the role(s) played.<br><br>* typeset	
**Competing Interests**<br><br>Use the instructions below to enter a competing interest statement for this submission. On behalf of all authors, disclose any competing interests that could be perceived to bias this work—acknowledging all financial support and any other relevant financial or non-financial competing interests.<br><br>This statement **will appear in the published article** if the submission is accepted. Please make sure it is accurate. View published research articles from *PLOS ONE* for specific examples.	The authors have declared that no competing interests exist.

**NO authors have competing interests**<br><br>Enter: *The authors have declared that no competing interests exist.*<br><br>**Authors with competing interests**<br><br>Enter competing interest details beginning with this statement:<br><br>*I have read the journal's policy and the authors of this manuscript have the following competing interests: [insert competing interests here]*<br><br>* typeset	
**Ethics Statement**<br><br>Enter an ethics statement for this submission. This statement is required if the study involved:<br><br>• Human participants<br>• Human specimens or tissue<br>• Vertebrate animals or cephalopods<br>• Vertebrate embryos or tissues<br>• Field research<br><br>Write "N/A" if the submission does not require an ethics statement.<br><br>General guidance is provided below. Consult the submission guidelines for detailed instructions. **Make sure that all information entered here is included in the Methods section of the manuscript.**	The University of Regina Research Ethics Board<br>REB#: 2018-026<br>Consent was obtained in writing.

Powered by Editorial Manager® and ProduXion Manager® from Aries Systems Corporation

**Format for specific study types**<br><br>**Human Subject Research (involving human participants and/or tissue)**<br>• Give the name of the institutional review board or ethics committee that approved the study<br>• Include the approval number and/or a statement indicating approval of this research<br>• Indicate the form of consent obtained (written/oral) or the reason that consent was not obtained (e.g. the data were analyzed anonymously)<br><br>**Animal Research (involving vertebrate animals, embryos or tissues)**<br>• Provide the name of the Institutional Animal Care and Use Committee (IACUC) or other relevant ethics board that reviewed the study protocol, and indicate whether they approved this research or granted a formal waiver of ethical approval<br>• Include an approval number if one was obtained<br>• If the study involved *non-human primates*, add *additional details* about animal welfare and steps taken to ameliorate suffering<br>• If anesthesia, euthanasia, or any kind of animal sacrifice is part of the study, include briefly which substances and/or methods were applied<br><br>**Field Research**<br><br>Include the following details if this study involves the collection of plant, animal, or other materials from a natural setting:<br>• Field permit number<br>• Name of the institution or relevant body that granted permission	
**Data Availability**<br><br>Authors are required to make all data underlying the findings described fully available, without restriction, and from the time of publication. PLOS allows rare exceptions to address legal and ethical concerns. See the PLOS Data Policy and FAQ for detailed information.	Yes - all data are fully available without restriction

Powered by Editorial Manager® and ProduXion Manager® from Aries Systems Corporation

A Data Availability Statement describing where the data can be found is required at submission. Your answers to this question constitute the Data Availability Statement and **will be published in the article**, if accepted.<br><br>**Important:** Stating 'data available on request from the author' is not sufficient. If your data are only available upon request, select 'No' for the first question and explain your exceptional situation in the text box.<br><br>Do the authors confirm that all data underlying the findings described in their manuscript are fully available without restriction?	
**Describe where the data may be found in full sentences. If you are copying our sample text, replace any instances of XXX with the appropriate details.**<br><br>• If the data are **held or will be held in a public repository**, include URLs, accession numbers or DOIs. If this information will only be available after acceptance, indicate this by ticking the box below. For example: *All XXX files are available from the XXX database (accession number(s) XXX, XXX.).*<br>• If the data are all contained **within the manuscript and/or Supporting Information files**, enter the following: *All relevant data are within the manuscript and its Supporting Information files.*<br>• If neither of these applies but you are able to provide **details of access elsewhere**, with or without limitations, please do so. For example:<br><br>*Data cannot be shared publicly because of [XXX]. Data are available from the XXX Institutional Data Access / Ethics Committee (contact via XXX) for researchers who meet the criteria for access to confidential data.*<br><br>*The data underlying the results presented in the study are available from (include the name of the third party*	All relevant data are within the manuscript and its Supporting Information files.

and contact information or URL).<br>• This text is appropriate if the data are owned by a third party and authors do not have permission to share the data.<br><br>* typeset	
Additional data availability information:	

Manuscript

Click here to access/download;Manuscript;SK BK HA manuscript 2020 08 26.docx

A Patient Oriented Research Approach to Assessing Patients' and Primary Care Physicians' Opinions on Trauma-Informed Care

Seint Kokokyi, Ph.D.[1], Bridget Klest, Ph.D.[2], and Hannah Anstey, M.A.[2]

[1]Department of Clinical Health Psychology, Max Rady College of Medicine, University of Manitoba

[2]Department of Psychology, University of Regina

Corresponding author: Seint Kokokyi, skokokyi@wrha.mb.ca

Abstract

Objective To gather patients' and primary care physicians' (PCP) opinions on T[illegible]uma-Informed Care (TIC) and to investigate the acceptability of recommendations developed by patient, family, and physician advisors.

Design Cross-sectional research survey design and patient engagement.

Setting Canada, 2017 to 2019.

Participants English-speaking adults and licensed PCPs residing in Canada.

Main outcome measures Participants were given a b[illegible]ery of questionnaires including a list of physician actions and a list of recommendations consistent with TIC.

Results Patients and PCPs viewed TIC as important. Both patients and PCPs rated the following recommendations as helpful and likely to positively impact patient care: physician training, online trauma resource centres, information pamphlets, the ability to extend appointment times, and tr[illegible]a clinical pathways. PCPs' responses were significantly more positive than patients' responses.

Conclusion TIC is important to patients and PCPs. Patients and PCPs believe changes to physician training, patient engagement, and systemic factors would be helpful and likely to positively impact patient care. Future research needs to be conducted to investigate whether these recommendations improve patient care.

Healthcare professionals who engage in trauma-informed care (TIC) understand and respond to the impact of trauma while fostering safety, trustworthiness, peer support, empowerment, collaboration, and cultural sensitivity (1). There have been several reviews of TIC and its implications in the healthcare system(2–5); however, there is variability in what is considered to constitute TIC in the provision of TIC training within the healthcare system, and in the literature discussing the need for TIC in healthcare practice. Further, there are concerns that TIC is simply good patient-centered care and that to implement specialized training and practice would be redundant (3,5,6). The literature has also neglected to assess consumers' (i.e., patients) and providers' (i.e., physicians) opinions on TIC, whether they believe TIC is important, and whether or not TIC is already part of the care they receive/deliver. Lastly, patient engagement, which helps to tailor research to fit patient needs (7), has been inadequate in previous studies of TIC.

Individuals are more likely to seek help for mental health problems from general physicians than from mental health professionals (e.g., counsellors, psychologists, psychiatrists; 8). The percentage of family physicians providing mental health care ranges from 40-53% depending on the population setting (urban or rural 9) and primary care physicians (PCPs) have identified that one-third of their caseload involves addressing patients' mental health concerns (10). Given the unique position that PCPs are in when it comes to mental health care, assessing both PCP and patient opinions of TIC is of the utmost importance.

The current study was conducted with involvement from patient, family, and physician advisors, and was comprised of two phases. Participants' opinions of TIC were examined in Phase 1. The research team met to discuss the results from Phase 1 and to share their own experiences with the healthcare system, as consumers, providers, and family members. From

these discussions, recommendations were made for Phase 2 regarding physician training, patient education, and systemic changes. The acceptability of these recommendations was then assessed quantitatively.

PHASE 1

To our knowledge, no research has assessed the opinions of healthcare consumers (i.e., patients) and providers (i.e., physicians) on TIC. Further, among the research that has investigated the implementation of TIC, the importance of TIC to consumers and providers and whether or not TIC was already being practiced has not been assessed. In an effort to narrow this gap, the opinions of patients and PCPs on TIC were explored in Phase 1. The general population (patient sample, henceforth referred to as patients) and PCPs (physician sample, herein referred to as physicians) in Canada were surveyed regarding their perspectives on TIC. Patients were asked how frequently they perceived receiving aspects of TIC and how important each of these aspects were to them. Similarly, physicians were asked how frequently they perceived delivering aspects of TIC and how important each of these aspects were to their practice. Phase 1 had three objectives: to investigate patient and physician opinions on the importance of aspects of TIC, to assess how often patients and physicians perceived receiving or delivering aspects of TIC, and to compare patient and physician responses.

Methods

This study received ethics approval by the University of Regina's Research Ethics Board. Phase 1 used a cross-sectional research survey design to understand patient and physician opinions on TIC. Patients were 18 years of age or older, lived in Canada, and could read English. Recruitment was conducted through Qualtrics Panel, which allowed for a sample that was an approximate representation of the Canadian population with regard to gender, province, and

ethnicity (11). Patients completed a demographics (i.e., age, gender, ethnicity, education) questionnaire and the TIC survey-patient version. PCPs in Canada were recruited for the physician sample using purposive and snowball sampling methods. Approximately 850 physicians were invited to participate in the study through email. Physicians were informed that a $20 donation (up to $1000) would be made to a patient advocacy organization for their participation in Phase 1. Physicians completed a set of demographic questions and the TIC survey-physician version.

In addition to the authors, the research team also consisted of two patient advisors, one family advisor, one PCP, and a volunteer research assistant who were actively involved throughout the research process. Each of the patient advisors experienced a personal history of trauma and the family advisor had a family member with a trauma history. The physician advisor for this study attended medical school and completed residency training in Canada and had been practicing as a family physician in Canada for several years.

Measure

The TIC survey, patient version and physician version, is a 29-item questionnaire that assesses the frequency and importance of TIC principles in primary care. This survey was developed for the current study based on behavioural aspects of TIC principles. These questions were piloted on university students as well as physicians in the community, and were refined in collaboration with patient and physician advisors. Patients and physicians were asked to rate the frequency with which they received (patients) or provided (physicians) the indicated services (“Never,” “Seldom,” “Occasionally,” “To a considerable degree,” and “Almost always”) and the importance of these services (“Not important,” “Slightly important,” “Moderately important,” “Important,” and “Very important”). Example questions include: “Make you feel welcome by

being warm and friendly, and using a welcoming tone of voice"; "Provide you with choices that fit your life circumstances for treatment preferences"; "Help you identify your strengths and skills that may facilitate management of your own care"; and "Ask you about your cultural beliefs of your health and illness, and how you prefer to treat yourself."

The TIC survey average frequency and importance scores were calculated for each subsample (patients and physicians) to yield the TIC Survey Average-Frequency and TIC Survey Average-Importance for each group. Subscale scores were calculated by averaging responses to sets of questions assessing each of the TIC principles (henceforth referred to as aspects of TIC). Subscales for aspects of TIC were Trauma (understanding of patients' past traumatic events and its impact on patients), Safety (creating an environment that is physically and emotionally safe for patients), Trust (building and maintaining trust with patients), Collaboration (leveling power between provider and patient), Empowerment (drawing on individual patients' strength and experiences), Peer Support (connection to other trauma survivors for support), and Cultural Sensitivity (cultural responsiveness including awareness of cultural stereotypes and biases; 1).

Repeated measures ANOVA was used to observe whether one aspect of TIC was favoured over another among patients and/or physicians. Additionally, within the physician subsample, if there were any discrepancies between importance ratings and frequency ratings, physicians were asked to check from a list of possible reasons to explain the disconnect. Patients and physicians were also given an opportunity to provide written responses about patient care.

Results

Two hundred and ninety-six patients completed the study. The average age was 46.3 years and 54.4% identified as female. Ethnicity and province of residence were proportional to the Canadian census. Sixty physicians completed the survey. Physicians' average age was 39.7

years, and the majority (61.4%) identified as female (36.8% male, and 1.8% "other"). Demographic information for patients and physicians can be found in Table 1.

A repeated measures ANOVA was used to compare the averages of the different TIC aspects among patients and physicians (Table 2). There were significant differences in how frequently patients perceived receiving TIC aspects and how important they viewed the same aspects. For both frequency and importance, the Trauma aspect was rated lowest by both patients and physicians. Significant differences were observed in which physician ratings were higher for how frequently they perceived delivering TIC aspects and how important they viewed these aspects compared to patients. Safety, Trust, and Collaboration aspects were rated higher than Trauma, Empowerment, Peer Support, and Cultural Sensitivity by both patients and physicians.

In regards to written responses, some patients commented on positive experiences with their PCPs, who they described as caring and understanding. Others provided suggestions related to listening, communication, and having more time. Physician comments about general care include themes related to listening, time, and having empathy

Discussion

Overall, physicians' perceptions of delivering TIC were higher than patients' perceptions of receiving TIC, and physicians viewed TIC as more important than patients. Inquiring about trauma history was the lowest rated TIC aspect among both patients and physicians. Patients and physicians may believe this type of screening is not relevant in a primary care setting. Given that trauma has been linked to numerous psychological and medical problems (12), screening for the impact of trauma instead of screening for trauma itself may be more beneficial (3), Physician knowledge of the impact of trauma can lead to appropriate referrals, but simply knowing about patients' trauma history may not be helpful.

Trust, Safety, and Collaboration were the highest rated aspects of TIC among patients and physicians. Past research has demonstrated that trust in physicians and patient-physician collaboration are associated with greater patient satisfaction and positive patient health outcomes (13–15). Without trust, patients may be less likely to share relevant information and may not follow through with treatment. Thus, physicians in this study may have already been aware of the importance of trust and collaboration through their own experience and rated it highly as a result. Physician responses regarding safety may be related to the growing patient safety culture in primary care (16–18). It is likely that physicians already have an interest in promoting a culture of safety and their responses reflected this interest.

PHASE 2

In Phase 1 both patients and physicians rated TIC as important for patient care; however, there was a discrepancy: patients did not perceive receiving TIC at the same level that physicians perceived administering it. Recommendations were discussed with patient, family, and physician advisors on how to close this gap between. After discussion with the advisors, the main recommendations were: physician training, booking longer appointment times, patient education, support groups for patients, and treatment planning. The aims of Phase 2 were to ask participants (patients and physicians) how helpful these recommendations would be and how likely they would be to use them if made available, and to compare patients' and physicians' responses.

Methods

Approval for Phase 2 was obtained from the University of Regina's Research Ethics Board. A cross sectional survey research design was used in Phase 2 to investigate the acceptability of recommendations developed by the research team, surveying a subsample of patients and physicians who had previously participated in Phase 1.

The patient and physician surveys used in Phase 2 were developed based on the results from Phase 1 and discussions with the advisors. Recommendations included on the surveys were physician training (i.e., TIC training, gender-related differences in trauma and healthcare, ethnicity-related disparities in trauma and healthcare, trauma and healthcare in marginalized groups, emotional skills, and self-compassion), billing mechanisms (i.e., to allow for booking ahead longer appointment times or to extend appointment times when situations arise unexpectedly), patient engagement (i.e., information pamphlets, peer support groups, and trauma resource centre), and clinical pathway with guidelines for assessment and treatment of trauma. Patients and physicians were asked how helpful they found each of the recommendations, about the likelihood that the recommendations would positively impact patient care, and about the likelihood that they would utilize these options if made available. For each of the recommendations on physician training, physician participants were asked on a five-point Likert scale how likely they would attend the training if it were online, if in person, if continuing medical education (CME) credit was offered, and if CME credit was not offered. They were also asked how many CME credit hours would be ideal.

Results

A total of 151 patients and 36 physicians from Phase 1 completed Phase 2 survey. Participants' demographic information can be found in Table 1. Table 3 outlines mean patient and physician responses for physician training. Patients and physicians indicated that it would be "moderately" to "very helpful" for physicians to receive training in TIC, gender related issues in trauma and healthcare, ethnicity related issues in trauma and healthcare, trauma and healthcare in marginalized groups, emotional skills, and self-compassion. There were no differences in physician and patient ratings across the different physician training recommendations. Patients

and physicians reported that all of the training would “likely” positively impact their patient care. TIC physician training was rated as more likely to positively impact care than training for gender related issues in trauma and healthcare. There were no differences between patients and physicians in how they responded to how helpful physician training in TIC would be or how likely this training would positively impact patient care. The likelihood that physicians would attend any of this training in person or online ranged between “neither likely nor unlikely” and “likely.” Physicians indicated they would be more likely to attend the training if CME credit was offered and the average ideal number of CME hours ranged between 2.16 to 3.26.

On average patients thought it would be “moderately helpful” to “very helpful” to have a mechanism for booking longer appointment times in advance, and they thought it would “likely” positively impact patient care. Patients thought it would be “moderately helpful” to “very helpful” to have a mechanism for extending appointment times should as situation come up unexpectedly, and they thought this recommendation would “likely” positively impact patient care. Physicians thought it would be “very helpful” to “extremely helpful” to have a mechanism for booking longer appointment times in advance and thought it would “likely” to “very likely” positively impact their patient care. They also thought it would be “very helpful” to “extremely helpful” to have a mechanism for extending appointment times should a situation come up unexpectedly and they indicated this would “likely” to “very likely” positively impact their patient care. Results from a repeated measures ANOVA (Table 4) showed a significant within-subjects effect such that patients and physicians found extending appointment times to be more helpful and more likely to positively impact patient care than booking ahead longer appointments, and between-subject effect such that physicians’ ratings were significantly higher than patients’ for all four ratings.

For all the pamphlets, patients indicated they would find them “moderately helpful” to “very helpful” and the likelihood they would refer to them ranged from “neither likely nor unlikely” to “likely” (Table 5). The pamphlets regarding “How Trauma Impacts Physical and Mental Health” and “Understanding PTSD and PTSD Treatment” received the highest ratings among patients and physicians. Physicians indicated these pamphlets would be “moderately helpful” to “extremely helpful” and the likelihood they would refer their patients to the pamphlets ranged from “neither likely nor unlikely” to “very likely” (Table 5). Physicians indicated these pamphlets would be “very likely” to “extremely likely” to refer patients to these pamphlets. Patients’ responses were significantly lower than physicians for the helpfulness of, and the likelihood of referring to, these pamphlets.

On average, patients rated each of the peer support groups to be “moderately helpful” and indicated they were “unlikely” to “neither likely nor unlikely” to attend either of the groups in person or online (Table 6). There were no differences in the likelihood of attending peer support groups in person or online for specific concerns, for general concerns, or for navigating the healthcare system. Physicians’ ratings of the helpfulness of peer support groups ranged from “moderately helpful” to “extremely helpful” and they indicated they were “likely” to refer their patients to in person or online groups (Table 6). For trauma survivors with specific concerns, physicians were more likely to refer their patients to an in-person group than an online group. However, there were no differences in the likelihood of physicians referring their patients for an in person or an online group for general concerns or for navigating the healthcare system as a trauma survivor. Patients rated peer support groups for trauma survivors with specific concerns and for navigating the healthcare system as less helpful than physicians (Table 6). For all three

peer support groups, patients' ratings of the likelihood of joining the groups in person or online were lower than physicians' ratings of the likelihood of referring their patients to these groups.

Patients responded that a trauma resource website would be more helpful than a hotline and they indicated they would be more likely to use a website than a hotline (Table 7). Physicians indicated both hotline and website trauma resource centres would be "very helpful" to "extremely helpful" and reported these resources would "likely" to "very likely" positively impact their patient care (Table 7). There were no differences in how helpful they found each kind of resources or in how likely they thought these recommendations would positively impact patient care. Physicians' ratings were higher than patients' ratings for this recommendation.

Patients indicated they would find a clinical pathway for treating trauma to be "moderately helpful" to "very helpful" and would "neither likely nor unlikely" to "likely" refer to this pathway (Table 8). Physicians indicated they would find this pathway to be "moderately helpful" to "very helpful" and would "likely" to "very likely" refer to this pathway (Table 8). Patients reported they would be less likely to refer to a clinical pathway for trauma than physicians.

Discussion

Both patients and physicians reported that physician training would be helpful and would likely positively impact patient care. Patients may have these views given their position at the receiving end of healthcare and their first-hand experience with physicians' competence and compassion. Physicians may be aware of their level of competence as well as their areas for improvement and thus may view training as a way to advance their knowledge. Past studies (19–22) showed it is feasible to provide brief physician TIC training with favourable outcomes, such

as increased patient-centeredness, knowledge about trauma and its impacts, and increased confidence in discussing trauma with patients.

One of the fundamental principles of TIC is sensitivity to cultural, historical, and gender-based issues (23). Understanding gender differences in healthcare including the power differential that exists between genders, differing levels of sensitivity to safety, and differing social roles, is integral to physicians creating spaces that are physically and emotionally safe for patients (24,25). Physician training in gender-based issues related to trauma and healthcare could help physicians recognize any gender-related ideologies, stereotypes, and inequity within the healthcare system they operate in. This in turn could help them provide better care to their patients. Training in culturally-sensitive care may provide physicians with the tools they need to integrate patients' cultural meaning of health and illness in their practice. Further, training could help physicians consider how patients' experiences of racism and discrimination influence their health, access to healthcare, and quality of life (26). Individuals belonging to multiple marginalized social categories experience more social obstacles than individuals who only belong to one category (27). Physician training on marginalized groups may allow physicians to understand the discriminatory experiences of patients with intersecting statuses of marginality and subsequently use this understanding to respond appropriately to patient needs.

Physician training aimed at increasing emotional intelligence and/or emotional skills could help physicians better understand and recognize emotional expression in themselves and in their patients (28–30). Emotional intelligence training could educate physicians on how to be more responsive to their patients.

Physician compassion has been associated with patient trust(31) and increased collaboration with their patients (32). Physicians who receive training in self-compassion have

an increased awareness of how their presence impacts patients and are more likely to provide nurturing care to their patients (33). Increasing physician compassion through self-compassion training could lead to better patient care, trust, and collaboration.

Physicians rated changes to billing mechanisms to be more helpful and more likely to positively impact patient care than patients. Patients may not understand how physicians are compensated or the mechanisms behind billing, and resultingly, may not think recommendations about billing are as important to their care. For both samples, having a mechanism in place to extend appointment time due to unexpected situations was perceived as more helpful and more likely to positively impact patient care than booking a longer appointment time in advance. It may be difficult to gauge how long patients will need for any given concern. When unexpected events come up requiring a longer appointment time, some physicians may grant patients extra time at the cost of delaying remaining appointments, which may have cumulative effect over the course of a day. Other physicians may not be able to provide patients with extra time and may end appointments sooner than what patients would like, causing patients to feel rushed and unsatisfied with the care they received. TIC training is unlikely to solve this scheduling problem; however, physicians should be fairly compensated for their time.

Physicians rated all patient engagement recommendations (i.e., information pamphlets, peer-support groups, and trauma resource centre), as more helpful and more likely to positively impact patient care than patients. Physicians may think of the recommendations as relevant to their patient population as a whole, with certain recommendations being relevant to certain patients. Therefore, physicians might view the recommendations as both helpful and positive as they can think of numerous patients who might benefit from them. In contrast, each individual

patient may only find certain recommendations to be personally relevant or beneficial and thus may rate the other recommendations as less helpful and impactful.

The information pamphlets “How Trauma Impacts Physical and Mental Health” and “Understanding PTSD and PTSD Treatment” received high ratings for how helpful and how likely they would be referred to by both patients and physicians. It may be beneficial for patients to have information on these topics as the lifetime exposure to one or more traumatic event in Canada has been estimated to be 75.9% (34). Patients’ ratings for the likelihood of referring to the other potential pamphlet topics were low, and thus it may not be as useful to have these pamphlets readily available.

While patients in this study reported they would find peer support groups moderately helpful, they indicated they were unlikely to attend any of the suggested groups, online or in person. Even though physicians may see benefits of these groups and would be willing to refer their patients, there may be little use if patients are unlikely to attend.

Patients indicated trauma resource websites would be more helpful and more likely to positively impact patient care than trauma resource hotlines. Research has shown that individuals use the Internet to seek health information (35–37). Benefits to online resources include increased access to care (38) and reduced travel costs (39). Online resources also offer confidentiality for individuals who have concerns about anonymity (40). Physicians reported that online and over the phone trauma resource centres would be similarly helpful and likely to positively impact patient care. Given the positive reception to this recommendation, development and implementation of comprehensive online psychological support programs for trauma survivors in Canada should be considered.

Physicians refer to clinical pathways to help with treatment planning (e.g., referrals, triage, available services) and clinical pathways help physicians understand available services (41). Physicians in this study rated clinical pathways for trauma care as more helpful and more likely to positively impact care than patients. Patients may not have an understanding of clinical pathways and their impact on healthcare. Patients may also not know what services exist. Physicians are able to provide choices to their patients when they know what resources are available. As there is currently no research on the impact of clinical pathways on TIC, this is an area for future research consideration.

GENERAL DISCUSSION

On average, patients and physicians found TIC to be important. Patients reported receiving aspects of TIC, but not nearly as frequently as physicians reported delivering them. While previous research has suggested that TIC may simply be good patient care given that its core components are safety, trust, collaboration, empowerment, and cultural sensitivity (42–44), TIC is unique as it involves understanding patients' experiences through a trauma lens.

The aim of this study was to investigate patients' and physicians' opinions of TIC by using a patient-oriented research approach. Overall, TIC was seen as important and already practiced in primary care; however, aspects related to Safety, Trust, and Collaboration were rated higher than Trauma, Empowerment, Peer Support, and Cultural Sensitivity. Using these results, patient, family, and physician advisors identified recommendations they thought would be helpful for patients and primary care physicians. These recommendations were in the form of physician training, patient engagement, and system factors.

To facilitate the provision of TIC, physicians should receive training in trauma-informed care, gender differences, ethnic disparities, marginalized groups, emotional skills, and self-

compassion. TIC physician training may provide physicians with a fundamental knowledge of trauma and its impact on individuals. Further, TIC training may help physicians learn how to foster a sense of safety, trust, empowerment, collaboration, and cultural sensitivity in their patients. Physician training in gender differences, ethnic disparities, and marginalized groups could provide physicians with knowledge on how trauma and health differs across groups. Further, this training could help physicians gain insight into how social roles and cultural norms influence patients' perceptions of safety and power dynamics. Emotional skills and self-compassion training could offer additional skills to help physicians understand and respond compassionately to patients' emotions.

Patients and physicians reported they would find the aforementioned physician trainings to be helpful and likely to positively impact patient care. Physicians indicated they would likely attend training if CME credit was offered. Healthcare regions/authorities and the Royal College of Physicians and Surgeons of Canada, which is responsible for developing CME programs (45), should consider developing and offering these kinds of training for physicians. To help incentivize physicians to seek this kind of training and to implement TIC in practice, physicians should be adequately compensated for extended appointment times and for providing counselling to their patients.

Information pamphlets and online trauma resource centres may provide patients with the education and support they need to help manage any emotional difficulties. Further, having access to accurate health information could help empower patients to make informed decisions (46,47) and could promote shared decision-making (46). Patients and physicians indicated that information pamphlets on “How trauma impacts physical and mental health” and “Understanding PTSD and PTSD treatment” would be helpful and they indicated that they would

likely refer to them (or refer their patients to them). While pamphlets on “Understanding PTSD and PTSD treatment” (48,49) already exist, provincial healthcare systems should work on improving accessibility of such documents for both physicians and patients through continued education on the resources available. While information on “How trauma impacts physical and mental health” is not currently available in a single document, one could easily be created and made readily accessible to physicians and patients.

Online trauma resource centres could provide patients with information to help facilitate understanding of their traumatic experiences and in turn foster a sense of empowerment. Trauma resource centres could help fulfill the TIC principle of empowerment. One already exists in Nova Scotia (50), but there are none at the national level. This may be a relatively low-cost recommendation to implement to increase patient engagement and awareness. Similarly, clinical pathways could be developed at a low cost, and help direct physicians to some of these programs as well as other resources in the community that promote patient wellness.

Strengths and limitations

TIC was developed based on research about service interventions for women survivors of violence with concurrent disorders (51) and has been implemented in healthcare (2–5). In this process, there has been little to no investigation of patients’ and physicians’ opinions on TIC. The present study attempted to fill a gap in the research by examining perspectives from two different stakeholder groups: patients and primary care physicians.

Another strength of this study was the active engagement of patient, family, and physician advisors throughout the research process using a patient-oriented research approach. Engaging patients makes research more accountable and transparent, helps provide new insights, and ensures research is relevant for patients (52). All the recommendations in Phase 2 were

directly relevant to patient care as they were formulated in conjunction with patient, family, and physician advisors. Furthermore, integrated knowledge translation (applying principles of knowledge translation to the entire research process) was applied throughout the duration of the research project as knowledge users (i.e. patients and physicians) were involved (53).

The patient sample in Phase 1 was representative of the Canadian population in regards to the gender, ethnicity, and province of residence. However, Canada's population is approximately 35 million (11) and the perspectives described in this study may not be shared by all in Canada. While efforts were made to recruit primary care physicians from across Canada, only physicians from six provinces and one territory participated. Considering there are 43,500 licensed physicians in family medicine/general practice (54), a sample size of 60 physicians is small and is likely not fully representative of family physicians practicing in Canada.

There could have been potential biases in physicians' responses. Although participants were informed that their responses would be de-identified for analysis, physicians were responding to questions about their own practice and may have responded in a socially desirable way as they may not have wanted to appear negative or uncaring. Approximately 850 physicians were individually invited to participate, but only 60 completed the Phase 1 survey. Perhaps physicians who participated were interested in the topic, found the topic important, and/or were already practicing some variation of TIC. This may possibly explain the positive responses to the survey items in both Phase 1 and 2.

Conclusion

Results from Phase 1 showed that both patients and physicians found TIC to be important. While physicians reported they were already delivering aspects of TIC to a considerable degree, patients indicated they were occasionally receiving them. These findings

suggest there is a need for TIC training as patients find TIC important but are not receiving it frequently. Recommendations on physician training, patient engagement, and system factors (i.e., billing for appointments and clinical pathways) that affect physicians' ability to provide TIC for their patients were investigated in Phase 2. Results showed these recommendations were acceptable to patients and physicians; however, further research is needed to investigate whether these recommendations improve patient care. Programs that implement these recommendations could be developed and subsequently evaluated to assess improvements in clinical outcomes. These findings suggest that most aspects of TIC are deemed important by patients and physicians. Furthermore, a number of cost-effective interventions to facilitate the implementation of TIC principles by physicians have been identified.

Acknowledgements

Special thanks to Ms. Tracy Kydd, Dr. John Fryters, Mrs. Hannelore Fryters, Dr. Crystal Ferguson, and Mr. Emiolio Filomeno for their involvement throughout the research process.

References

1. Substance Abuse and Mental Health Services Administration. SAMHSA's Concept of Trauma and Guidance for a Trauma-Informed Approach. 2014;27.

2. Anyikwa VA. Trauma-Informed Approach to Survivors of Intimate Partner Violence. J Evid-Inf Soc Work. 2016 02;13(5):484–91.

3. Machtinger EL, Cuca YP, Khanna N, Rose CD, Kimberg LS. From Treatment to Healing: The Promise of Trauma-Informed Primary Care. Womens Health Issues. 2015 May 1;25(3):193–7.

4. Oral R, Ramirez M, Coohey C, Nakada S, Walz A, Kuntz A, et al. Adverse childhood experiences and trauma informed care: the future of health care. Pediatr Res. 2016 Jan;79(1–2):227–33.

5. Raja S, Hasnain M, Hoersch M, Gove-Yin S, Rajagopalan C. Trauma informed care in medicine: current knowledge and future research directions. Fam Community Health. 2015 Sep;38(3):216–26.

6. Berliner L, Kolko DJ. Trauma Informed Care: A Commentary and Critique. Child Maltreat. 2016 Jan 9;21(2):168–72.

7. Bishop AC, Elliott MJ, Cassidy C. Moving patient-oriented research forward: thoughts from the next generation of knowledge translation researchers. Res Involv Engagem [Internet]. 2018 Aug 1 [cited 2019 Nov 2];4. Available from: https://www.ncbi.nlm.nih.gov/pmc/articles/PMC6069714/

8. Komiti A, Judd F, Jackson H. The influence of stigma and attitudes on seeking help from a GP for mental health problems: a rural context. Soc Psychiatry Psychiatr Epidemiol. 2006 Sep;41(9):738–45.

9. Xierali IM, Tong ST, Petterson SM, Puffer JC, Phillips RL, Bazemore AW. Family physicians are essential for mental health care delivery. J Am Board Fam Med JABFM. 2013 Apr;26(2):114–5.

10. Abed Faghri NM, Boisvert CM, Faghri S. Understanding the expanding role of primary care physicians (PCPs) to primary psychiatric care physicians (PPCPs): enhancing the assessment and treatment of psychiatric conditions. Ment Health Fam Med. 2010 Mar;7(1):17–25.

11. Statistics Canada. Census Profile, 2016 Census. Statistics Canada Catalogue No. 98-508-X2016001 [Internet]. 2018 [cited 2018 Feb 12]. Available from: http://www12.statcan.gc.ca/census-recensement/2016/dp-pd/prof/index.cfm?Lang=E

12. Felitti VJ, Anda RF. Therelationship of adverse childhood experiences to adult medical disease, psychiatric disorders and sexual behavior: implications for healthcare. In: Lanius RA, Vermetten E, Pain C, editors. Cambridge: Cambridge University Press; 2010. p. 77–87. (The Impact of Early Life Trauma on Health and Disease: The Hidden Epidemic).

13. Arbuthnott A, Sharpe D. The effect of physician–patient collaboration on patient adherence in non-psychiatric medicine. Patient Educ Couns. 2009 Oct;77(1):60–7.

14. Fuertes JN, Mislowack A, Bennett J, Paul L, Gilbert TC, Fontan G, et al. The physician–patient working alliance. Patient Educ Couns. 2007 Apr;66(1):29–36.

15. Hall MA, Dugan E, Zheng B, Mishra AK. Trust in Physicians and Medical Institutions: What Is It, Can It Be Measured, and Does It Matter? Milbank Q. 2001 Dec;79(4):613–39.

16. Verbakel NJ, Langelaan M, Verheij TJM, Wagner C, Zwart DLM. Improving Patient Safety Culture in Primary Care: A Systematic Review. J Patient Saf. 2016 Sep;12(3):152.

17. Verbakel NJ, Van Melle M, Langelaan M, Verheij TJM, Wagner C, Zwart DLM. Exploring patient safety culture in primary care. Int J Qual Health Care. 2014 Dec 1;26(6):585–91.

18. Weaver SJ, Lubomksi LH, Wilson RF, Pfoh ER, Martinez KA, Dy SM. Promoting a Culture of Safety as a Patient Safety Strategy: A Systematic Review. Ann Intern Med. 2013 Mar 5;158(5_Part_2):369.

19. Goldstein E, Murray-García J, Sciolla AF, Topitzes J. Medical Students' Perspectives on Trauma-Informed Care Training. Perm J [Internet]. 2018 Jan 18 [cited 2019 Mar 29];22. Available from: https://www.ncbi.nlm.nih.gov/pmc/articles/PMC5798942/

20. Green BL, Saunders PA, Power E, Dass-Brailsford P, Schelbert KB, Giller E, et al. Trauma-Informed Medical Care: A CME Communication Training for Primary Care Providers. Fam Med. 2015 Jan;47(1):7–14.

21. Hoysted C, Jobson L, Alisic E. A pilot randomized controlled trial evaluating a web-based training program on pediatric medical traumatic stress and trauma-informed care for emergency department staff. Psychol Serv. 2019 Feb;16(1):38–47.

22. Weiss D, Kassam-Adams N, Murray C, Kohser KL, Fein JA, Winston FK, et al. Application of a Framework to Implement Trauma-Informed Care Throughout a Pediatric Health Care Network: J Contin Educ Health Prof. 2017;37(1):55–60.

23. Substance Abuse and Mental Health Services Administration. Trauma-Informed Care in Behavioral Health Services. Treatment Improvement Protocol (TIP) Series 57. HHS PublicationNo. (SMA) 13-4801. Rockville, MD: Substance Abuse and Mental Health Services Administration, 2014; 2014.

24. Pattyn E, Verhaeghe M, Bracke P. The gender gap in mental health service use. Soc Psychiatry Psychiatr Epidemiol. 2015 Jul 1;50(7):1089–95.

25. Slaunwhite AK. The Role of Gender and Income in Predicting Barriers to Mental Health Care in Canada. Community Ment Health J N Y. 2015 Jul;51(5):621–7.

26. Browne AJ, Varcoe CM, Wong ST, Smye VL, Lavoie J, Littlejohn D, et al. Closing the health equity gap: evidence-based strategies for primary health care organizations. Int J Equity Health. 2012 Dec;11(1):59.

27. Jackson JW, Williams DR, VanderWeele TJ. Disparities at the intersection of marginalized groups. Soc Psychiatry Psychiatr Epidemiol. 2016 Oct 1;51(10):1349–59.

28. Dugan JW, Weatherly RA, Girod DA, Barber CE, Tsue TT. A Longitudinal Study of Emotional Intelligence Training for Otolaryngology Residents and Faculty. JAMA Otolaryngol Neck Surg. 2014 Aug 1;140(8):720–6.

29. Riess H, Kelley JM, Bailey RW, Dunn EJ, Phillips M. Empathy Training for Resident Physicians: A Randomized Controlled Trial of a Neuroscience-Informed Curriculum. J Gen Intern Med. 2012 Oct;27(10):1280–6.

30. Satterfield JM, Hughes E. Emotion skills training for medical students: a systematic review. Med Educ. 2007;41(10):935–41.

31. Mechanic D, Meyer S. Concepts of trust among patients with serious illness. Soc Sci Med. 2000 Sep 1;51(5):657–68.

32. Lown BA, Hanson JL, Clark WD. Mutual influence in shared decision making: a collaborative study of patients and physicians. Health Expect [Internet]. 2009 Feb [cited 2019 May 24]; Available from: http://doi.wiley.com/10.1111/j.1369-7625.2008.00525.x

33. Schneider S, Kingsolver K, Rosdahl J. Physician Coaching to Enhance Well-being: A Qualitative Analysis of a Pilot Intervention. EXPLORE. 2014 Nov 1;10(6):372–9.

34. Van Ameringen MV, Mancini C, Patterson B, Boyle MH. Post-Traumatic Stress Disorder in Canada. CNS Neurosci Ther. 2008;14(3):171–81.

35. Burns JM, Davenport TA, Durkin LA, Luscombe GM, Hickie IB. The internet as a setting for mental health service utilisation by young people. Med J Aust. 2010;192(S11):S22–6.

36. Chou WS, Liu B, Post S, Hesse B. Health-related Internet use among cancer survivors: data from the Health Information National Trends Survey, 2003–2008. J Cancer Surviv. 2011 Sep;5(3):263–70.

37. Fox S, Duggan M. Health Online 2013 [Internet]. Pew Research Center; 2013 [cited 2019 Apr 26] p. 1–55. Available from: https://www.pewinternet.org/wp-content/uploads/sites/9/media/Files/Reports/PIP_HealthOnline.pdf

38. Dorsey ER, Topol EJ. State of Telehealth. N Engl J Med. 2016 Jul 14;375(2):154–61.

39. Moffatt JJ, Eley DS. The reported benefits of telehealth for rural Australians. Aust Health Rev Collingwood. 2010 Aug;34(3):276–81.

40. Sinclair C, Holloway K, Riley G, Auret K. Online Mental Health Resources in Rural Australia: Clinician Perceptions of Acceptability. J Med Internet Res. 2013 Sep 5;15(9):e193.

41. Cappelli M. Creating integrated pathways to care between primary care and community basec child and youth mental health services. 20th Canadian Collaborative Mental Health Conference; 2019 May 10; Richmond, BC.

42. Barry MJ, Edgman-Levitan S. Shared Decision Making — The Pinnacle of Patient-Centered Care. N Engl J Med. 2012 Mar 1;366(9):780–1.

43. Maizes V, Rakel D, Niemiec C. Integrative Medicine and Patient-Centered Care. EXPLORE. 2009 Sep 1;5(5):277–89.

44. Sammer CE, Lykens K, Singh KP, Mains DA, Lackan NA. What is Patient Safety Culture? A Review of the Literature. J Nurs Scholarsh. 2010;42(2):156–65.

45. Royal College of Physicians and Surgeons of Canada. The Royal College of Physicians and Surgeons of Canada :: The Maintenance of Certification (MOC) Program [Internet]. 2019 [cited 2019 May 23]. Available from: http://www.royalcollege.ca/rcsite/cpd/maintenance-of-certification-program-e

46. Dixon-Woods M. Writing wrongs? An analysis of published discourses about the use of patient information leaflets. Soc Sci Med. 2001 May 1;52(9):1417–32.

47. Garner M, Ning Z, Francis J. A framework for the evaluation of patient information leaflets. Health Expect. 2012;15(3):283–94.

48. Centre for Addiction and Mental Health. Posttraumatic Stress [Internet]. 2006. Available from: https://www.camh.ca/-/media/files/guides-and-publications/posttraumatic-stress.pdf

49. National Center for PTSD. Understanding PTSD and PTSD treatment [Internet]. 2019. Available from: https://www.ptsd.va.gov/publications/print/understandingptsd_booklet.pdf

50. The Nova Scotia Trauma Informed Network. The Nova Scotia Trauma Informed Network: Decolonizing violence and trauma informed research, knowledge translation and transfer, policy and practice [Internet]. 2019 [cited 2019 Nov 23]. Available from: https://www.novascotiatraumainformednetwork.org/

51. Wilson C, Pence DM, Conradi L. Trauma-Informed Care. In National Association of Social Workers and Oxford University Press USA Factory; 2013. (Encyclopedia of Social Work). Available from: http://socialwork.oxfordre.com/10.1093/acrefore/9780199975839.001.0001/acrefore-9780199975839-e-1063

52. Canadian Institutes of Health Research. Evaluation of the Strategy for Patient-Oriented Research [Internet]. 2016 [cited 2019 Mar 25] p. 97. Available from: http://www.cihr-irsc.gc.ca/e/49937.html

53. Canadian Institutes of Health Research. Guide to Knowledge Translation Planning at CIHR: Integrated and End-of-Grant Approaches - CIHR [Internet]. Her Majesty the Queen in Right of Canada; 2012 [cited 2019 May 14]. Available from: http://www.cihr-irsc.gc.ca/e/documents/kt_lm_ktplan-en.pdf

54. Canadian Medical Association. Canadian physician data: Number and percent by specialty and sex, Canada 2018 [Internet]. 2018 [cited 2019 May 14]. Available from: https://www.cma.ca/sites/default/files/pdf/Physician%20Data/06-spec-sex.pdf

Table 1. Participants' Demographic Information

Factor	*M* (*SD*) / Proportion*			
	Patients		Physicians	
	Phase 1	Phase 2	Phase 1	Phase 2
N	296	151	60	36
Age	46.28 (14.6)	49.2 (13.1)	39.70 (10.6)	39.5 (10.3)
Gender				
Female	54.4 %	51.0 %	61.4%	62.2 %
Male	44.9%	48.3 %	36.8%	37.8 %
Other	0%	0%	1.8%	2.8%
Ethnicity				
White	43.6%	45.0 %	66.7%	63.9 %
Aboriginal	14.9%	11.9 %	1.7%	0%
Black	4.4%	3.3 %	0%	0%
South Asian	6.8%	7.9%	5.0%	5.6%
East Asian	17.9%	23.2%	10.0%	11.1%
Southeast Asian	1.4%	0%	5.0%	5.6%
Filipino	2.0%	1.3%	0%	0%
Arab	0.3%	0.7%	3.3%	5.6%
West Asian	1.0%	0.7%	0%	0%
Latin American	2.0%	0.7%	3.3%	2.8
Multiracial	4.4%	4.0%	5.0%	5.6
Population Setting				
Urban	77.4%	81.5 %	71.7%	75.7 %
Rural	18.9%	17.2 %	28.3%	24.3 %
First Nations Band	3.4%	1.3 %	1.7%	0%
Province or Territory				
British Columbia	15.2%	15.2 %	8.3%	10.8 %
Alberta	9.1%	7.9 %	45.0%	45.9 %
Saskatchewan	10.5%	10.6 %	20.0%	21.6 %
Manitoba	4.7%	4.0 %	0%	0%
Ontario	34.5%	38.4 %	16.7%	18.9 %
Quebec	7.4%	7.3 %	3.3%	0%
New Brunswick	3.4%	2.6 %	1.7%	0%
Nova Scotia	4.7%	4.6 %	0%	0%
Prince Edward Island	3.0%	2.6 %	0%	0%
Newfoundland and Labrador	4.7%	4.6 %	0%	0%
Yukon	1.7%	0%	0%	0%
Northwest Territories	0.3%	0.7 %	0%	0%
Nunavut	0.7%	1.3 %	3.3%	2.7 %

* Numbers may not add up to 100% as not all participants answered every question.

Table 2. Repeated measures ANOVA for patients' and physicians' opinion on aspects of Traum[illegible]formed Care

Groups	Trauma *M* (*SD*)	Safety *M* (*SD*)	Trust *M* (*SD*)	Collaboration *M* (*SD*)	Empower *M* (*SD*)	Peer Support *M* (*SD*)	Culture *M* (*SD*)	Multivar[illegible]est	Within-Subject Effects	Between-Subjects Effects
**Frequency Ratings**										
Patients	2.53 (1.38)	3.92 (1.24)	4.01 (1.22)	3.90 (1.22)	3.67 (1.31)	3.48 (1.48)	3.20 (1.41)	*TIC Factors:*<br>Wilks' Λ=.66<br>F(6,338)=29.05**<br>Partial η^2=.34<br><br>*Interaction:*<br>Wilks' Λ=.91<br>F(6,338)=5.38**<br>Partial η^2=.09	*TIC Factors:*<br>F(3.48,1190.71)=63.92**<br>Partial η^2=.16<br><br>*Interaction:*<br>F(3.47, 1190.71)=6.86**<br>Partial η^2=.02	*Patient/Physician*<br>F(1,343)=26.69**<br>Partial η^2=.07
Physicians	3.85 (.76)	4.57 (.43)	4.63 (.37)	4.60 (.31)	4.15 (.61)	4.20 (.71)	4.03 (.70)			
**Importance Ratings**										
Patients	3.01 (1.34)	3.85 (1.13)	3.98 (1.13)	3.89 (1.12)	3.74 (1.19)	3.58 (1.29)	3.26 (1.35)	*TIC Factors:*<br>Wilks' Λ=.81<br>F(6,336)=13.59**<br>Partial η^2=.20<br><br>*Interaction:*<br>Wilks' Λ=.95<br>F(6,336)=3.10<br>Partial η^2=.05	*TIC Factors:*<br>F(3.44,1172.41)=31.03**<br>Partial η^2=.08<br><br>*Interaction:*<br>F(3.44,1172.41)=5.66**<br>Partial η^2=.02	*Patient/Physician*<br>F(1,341)=43.65**<br>Partial η^2=.11
Physicians	4.37 (.77)	4.69 (.42)	4.73 (.35)	4.72 (.29)	4.49 (.61)	4.47 (.78)	4.33 (.76)			

Note. Helpfulness scores: 1 = Not at all helpful, 2 = Slightly helpful, 3 = Moderately helpful, 4 = Very helpful, and 5 = Extremely helpful. Likelihood scores: 1 = Very unlikely, 2 = Unlikely, 3 = Neither likely nor unlikely, 4 =Likely, and 5 =Very likely.

* $p \leq .01$.

** $p \leq .001$.

Table 3. Repeated measures ANOVA for patients' and physicians' opinion on physician training

Groups	TIC<br>*M* (*SD*)	Gender issues<br>*M* (*SD*)	Ethnic issues<br>*M* (*SD*)	Marginalized groups<br>*M* (*SD*)	Emotions<br>*M* (*SD*)	Self-compassion<br>*M* (*SD*)	Multivariate test	Within-Subject Effects	Between-Subjects Effects
**Helpfulness Ratings**									
Patients	3.72 (1.06)	3.76 (1.06)	3.67 (1.14)	3.65 (1.23)	3.82 (1.16)	3.81 (1.09)	*Training:*<br>Wilks' Λ=.94<br>F(5,165)=1.95<br>Partial η^2=.06<br><br>*Interaction:*<br>Wilks' Λ=.87<br>F(5,165)=4.88**<br>Partial η^2=.13	*Physician Training:*<br>F(4.13,698.67)=2.23<br>Partial η^2=.01<br><br>*Interaction:*<br>F(4.13,698.67)=2.95**<br>Partial η^2=.04	*Patient/Physician*<br>F(1,169)=.11<br>Partial η^2<.001
Physicians	3.90 (.923)	3.67 (1.06)	4.13 (.90)	3.97 (1.00)	3.43 (1.28)	3.70 (1.12)			
**Likelihood Ratings**									
Patients	3.71 (.92)	3.45 (1.15)	3.43 (1.13)	3.45 (1.11)	3.75 (1.02)	3.74 (.98)	*Training:*<br>Wilks' Λ=.90<br>F(5,165)=3.69*<br>Partial η^2=.10<br><br>*Interaction:*<br>Wilks' Λ=.94<br>F(5,165)=2.26<br>Partial η^2=.06	*Physician Training:*<br>F(3.84,648.06)=3.46*†<br>Partial η^2=.02<br>*Interaction:*<br>F(3.84,648.06)=3.84<br>Partial η^2=.02	*Patient/Physician*<br>F(1,169)=1.65<br>Partial η^2=.01
Physicians	4.03 (.93)	3.60 (1.25)	4.00 (1.17)	3.77 (1.19)	3.83 (1.12)	3.67 (1.12)			

Note. Helpfulness scores: 1 = Not at all helpful, 2 = Slightly helpful, 3 = Moderately helpful, 4 = Very helpful, and 5 = Extremely helpful. Likelihood scores: 1 = Very unlikely, 2 = Unlikely, 3 = Neither likely nor unlikely, 4 =Likely, and 5 =Very likely.

† TIC training was rated as more likely to positively impact patient care than training for gender-related issues (*p*=.002).

* $p \leq .01$.

** $p \leq .001$.

Table 4. Repeated measures ANOVA results comparing patients' and physicians' responses for booking longer appointments ahead of time and extending appointment times unexpectedly

Factor	Patients		Physicians		Multivariate test	Within-Subject Effects	Between-Subjects Effects
	Booking longer appointments ahea[illegible] of time *M* (*SD*)	Extending Appointment times Unexpectedly *M* (*SD*)	Booking longer appoints ahead of time *M* (*SD*)	Extending Appointment times Unexpectedly *M* (*SD*)			
Helpfulness	3.59 (1.13)	3.81 (1.06)	3.04 (.97)	4.36 (.76)	Wilks' Λ=.935<br>$F(1,185)=12.86^{**}$<br>Partial η^2=.065	$F(1,185)=12.86^{**}$<br>Partial η^2=.065	$F(1,185)=14.14^{**}$<br>Partial η^2=.071
Likelihood	3.70 (1.06)	3.82 (1.05)	4.36 (.87)	4.58 (.65)	Wilks' $\Lambda=.926^{**}$<br>$F(1,185)$=14.75<br>Partial η^2=.074	$F(1,185)=14.75^{**}$<br>Partial η^2=.074	

Note. Helpfulness scores: 1 = Not at all helpful, 2 = Slightly helpful, 3 = Moderately helpful, 4 = Very helpful, and 5 = Extremely helpful. Likelihood scores: 1 = Very unlikely, 2 = Unlikely, 3 = Neither likely nor unlikely, 4 =Likely, and 5 =Very likely.

* $p\leq .01$.

** $p\leq .001$

Table 5. Repeated measures ANOVA for patients' and physicians' opinion on information pamphlets

Information Pamphlet Topic	Patients *M* (*SD*)	Physicians *M* (*SD*)	Multivariate test	Within-Subject Effects	Between-Subjects Effects
**Helpfulness Ratings**					
Information on Different Kinds of Trauma and How to Cope with Trauma	3.26 (1.30)	3.85 (.78)	*Topics:*<br>Wilks' Λ=.80<br>$F(9,160)=4.51^{**}$<br>Partial η^2=.20<br><br>*Interaction:*<br>Wilks' Λ=.87<br>$F(9,160)=2.78^{*}$<br>Partial η^2=.14	*Topics:*<br>$F(6.72,1129.39)=5.12^{**}$<br>Partial η^2=.03<br><br>*Interaction:*<br>$F(6.72,1129.39)=3.25^{*}$<br>Partial η^2=.02	*Patient/Physician*<br>$F(1,168)=8.93^{*}$<br>Partial η^2=.05
How Trauma Impacts Physical and Mental Health	3.44 (1.30)	4.15 (.74)			
Understanding PTSD and PTSD Treatment	3.40 (1.32)	4.06 (.74)			
How Trauma Affects Relationships	3.38 (1.35)	3.94 (.78)			
Disasters and Traumatic Loss	3.26 (1.37)	3.59 (.96)			
Traumatic Stress and Substance Abuse Problems	3.30 (1.37)	4.03 (.67)			
Intimate Partner Violence	3.03 (1.44)	4.18 (.67)			
Trauma as a Result of Medical Errors	3.10 (1.45)	3.47 (1.05)			
When a Friend or Loved One has been Traumatized	3.27 (1.36)	3.68 (.94)			
Trauma Information for Parents	3.19 (1.43)	3.97 (.72)			
**Likelihood Ratings**					
Information on Different Kinds of Trauma and How to Cope with Trauma	3.20 (1.30)	4.00 (.66)	*Topics:*<br>Wilks' Λ=.78<br>$F(9,160)=5.04^{**}$<br>Partial η^2=.22<br><br>*Interaction:*<br>Wilks' Λ=.85<br>$F(5,160)=3.06^{*}$<br>Partial η^2=.15	*Topics:*<br>$F(6.75,1133.09)=6.51^{**}$<br>Partial η^2=.04<br><br>*Interaction:*<br>$F(6.75,1133.09)=3.59^{**}$<br>Partial η^2=.02	*Patient/Physician*<br>$F(1,168)=11.55^{**}$<br>Partial η^2=.07
How Trauma Impacts Physical and Mental Health	3.34 (1.27)	4.18 (.64)			
Understanding PTSD and PTSD Treatment	3.30 (1.35)	4.09 (.58)			
How Trauma Affects Relationships	3.25 (1.40)	3.97 (.68)			
Disasters and Traumatic Loss	3.22 (1.33)	3.58 (.94)			
Traumatic Stress and Substance Abuse Problems	3.20 (1.38)	4.09 (.68)			
Intimate Partner Violence	3.01 (1.38)	4.15 (.71)			
Trauma as a Result of Medical Errors	3.01 (1.42)	3.45 (1.06)			
When a Friend or Loved One has been Traumatized	3.15 (1.35)	3.73 (.98)			
Trauma Information for Parents	3.08 (1.46)	3.91 (.77)			

Note. Helpfulness scores: 1 = Not at all helpful, 2 = Slightly helpful, 3 = Moderately helpful, 4 = Very helpful, and 5 = Extremely helpful. Likelihood scores: 1 = Very unlikely, 2 = Unlikely, 3 = Neither likely nor unlikely, 4 =Likely, and 5 =Very likely.

* $p \leq .01$.

** $p \leq .001$.

Table 6. Descriptive statistics for patients' and physicians' responses to the helpfulness of peer support groups for trauma survivors and the likelihood of either joining or referring patients to in person and online groups.

Support Group Type	Helpfulness Score *M* (*SD*)	Likelihood Score		*t*-Test Scores comparing likelihood to attend groups in person or online
		In Person Support Group *M* (*SD*)	Online Support Group *M* (*SD*)	
	**Patients**			
Specific Concerns	3.19 (1.42)	2.30 (1.33)	2.49 (1.40)	*t*(143)=-2.26, *p*=.025, Cohen's *d*=.148
General Concerns	3.12 (1.38)	2.41 (1.32)	2.52 (1.37)	*t*(143)=-1.60, *p*=.112, Cohen's *d*=.098
Navigating the Healthcare System	3.07 (1.44)	2.34 (1.29)	2.48 (1.37)	*t*(139)=-1.86, *p*=.065, Cohen's *d*=.118
**Physicians**				
Specific Concerns	4.35 (.77)	4.42 (.75)	3.79 (1.11)	*t*(32)=3.29, *p*=.002, Cohen's *d*=.671
General Concerns	3.76 (1.08)	3.82 (.98)	3.58 (1.09)	*t*(32)=1.39, *p*=.174, Cohen's *d*=.234
Navigating the Healthcare System	3.76 (.82)	3.76 (.94)	3.64 (.93)	*t*(32)=.751, *p*=.458, Cohen's *d*=.130

Note. Helpfulness scores: 1 = Not at all helpful, 2 = Slightly helpful, 3 = Moderately helpful, 4 = Very helpful, and 5 = Extremely helpful. Likelihood scores: 1 = Very unlikely, 2 = Unlikely, 3 = Neither likely nor unlikely, 4 =Likely, and 5 =Very likely.

Table 7. Repeated measures ANOVA to investigate patients' and physicians' responses to hotline and website trauma resource centre

Groups	Helpfulness				Likelihood to use/refer			
	Hotline *M* (*SD*)	Website *M* (*SD*)	Multivariate test	Within-Subject Effects	Hotline *M* (*SD*)	Website *M* (*SD*)	Multivariate test	Within-Subject Effects
Patients	3.43 (1.25)	3.68 (1.11)	Wilks' Λ=.869 F(1,144)=21.77** Partial η^2=.131	F(1,144)=21.77** Partial η^2=.131	2.95 (1.29)	3.50 (1.21)	Wilks' Λ=.748 F(1,144)=48.45** Partial η^2=.252	F(1,144)=48.45** Partial η^2=.252
Physicians	4.24 (1.01)	4.35 (.88)	Wilks' Λ=.788 F(1,33)=8.87* Partial η^2=.212	F(1,33)=8.87* Partial η^2=.212	4.38 (.85)	4.56 (.82)	Wilks' Λ=.933 F(1,33)=2.36 Partial η^2=.067	F(1,33)=2.36 Partial η^2=.067

Note. Helpfulness scores: 1 = Not at all helpful, 2 = Slightly helpful, 3 = Moderately helpful, 4 = Very helpful, and 5 = Extremely helpful. Likelihood scores: 1 = Very unlikely, 2 = Unlikely, 3 = Neither likely nor unlikely, 4 =Likely, and 5 =Very likely.

* $p \leq .01$.

** $p \leq .001$.

Table 8. Independent t-tests comparing patients' and physicians' scores on clinical pathway and trauma resource centres

Category	Recommendations	Helpfulness Scores			Likelihood Scores		
		Patients *M* (*SD*)	Physicians *M* (*SD*)	t-Test scores	Patients *M* (*SD*)	Physicians *M* (*SD*)	t-Test scores
Clinical Pathway	Clinical Pathway for Treating Trauma	3.18 (1.27)	3.79 (1.11)	$t(177)=-2.53$ Cohen's d = .506	3.28 (1.23)	4.06 (.90)	$t(178)=-3.44^{**}$ Cohen's d =.725
Resource Centre	Hotline	3.44 (1.23)	4.24 (1.02)	$t(180)=-3.47^{**}$ Cohen's d =.700	2.92 (1.30)	4.38 (.85)	$t(180)=-6.27^{**}$ Cohen's d =1.334
	Website	3.68 (1.11)	4.35 (.88)	$t(178)=-3.27^{**}$ Cohen's d = .665	3.49 (1.22)	4.56 (.82)	$t(181)=-4.87^{**}$ Cohen's d =1.029

Note. Positive *t*-scores indicate higher patient ratings than physicians. Helpfulness scores: 1 = Not at all helpful, 2 = Slightly helpful, 3 = Moderately helpful, 4 = Very helpful, and 5 = Extremely helpful. Likelihood scores: 1 = Very unlikely, 2 = Unlikely, 3 = Neither likely nor unlikely, 4 =Likely, and 5 =Very likely.

* $p \leq .01$.

** $p \leq .001$.
