# Supplementary material for: A patient-oriented research approach to assessing patients’ and primary care physicians’ opinions on trauma-informed care
Source: PLoS One. 2021 Jul 9;16(7):e0254266. doi: 10.1371/journal.pone.0254266 (PMC8270182; doi:10.1371/journal.pone.0254266)
Supplement: Attachment — Submitted filename: Response to Reviewers.docx [file pone.0254266.s004.docx]

1. Please ensure that your manuscript meets PLOS ONE's style requirements, including those for file naming. The PLOS ONE style templates can be found at

<https://journals.plos.org/plosone/s/file?id=wjVg/PLOSOne_formatting_sample_main_body.pdf> and

<https://journals.plos.org/plosone/s/file?id=ba62/PLOSOne_formatting_sample_title_authors_affiliations.pdf>

*Thank you for the links. We hope the formatting now meets PLOS ONE’s style requirements*

2. Please include additional information regarding the survey or questionnaire used in the study and ensure that you have provided sufficient details that others could replicate the analyses. For instance, if you developed a questionnaire as part of this study and it is not under a copyright more restrictive than CC-BY, please include a copy, in both the original language and English, as Supporting Information.
Furthermore, in the methods section, please address the following:
- a description of any inclusion/exclusion criteria that were applied to participant recruitment.
- a description of how participants were recruited.

*We have included the survey questions used as part of the Supporting Information and additional information on participant inclusion/exclusion criteria and recruitment methods have been included.*

3.We note that the grant information you provided in the ‘Funding Information’ and ‘Financial Disclosure’ sections do not match.

 When you resubmit, please ensure that you provide the correct grant numbers for the awards you received for your study in the ‘Funding Information’ section.

*We have ensured the correct grant numbers for the award received for our study.*

Reviewers' comments:

Reviewer's Responses to Questions

**Comments to the Author**

1. Is the manuscript technically sound, and do the data support the conclusions?

The manuscript must describe a technically sound piece of scientific research with data that supports the conclusions. Experiments must have been conducted rigorously, with appropriate controls, replication, and sample sizes. The conclusions must be drawn appropriately based on the data presented.

Reviewer #1: Yes

Reviewer #2: Yes

 2. Has the statistical analysis been performed appropriately and rigorously?

 Reviewer #1: I Don't Know

Reviewer #2: Yes

 3. Have the authors made all data underlying the findings in their manuscript fully available?

The [PLOS Data policy](http://www.plosone.org/static/policies.action#sharing) requires authors to make all data underlying the findings described in their manuscript fully available without restriction, with rare exception (please refer to the Data Availability Statement in the manuscript PDF file). The data should be provided as part of the manuscript or its supporting information, or deposited to a public repository. For example, in addition to summary statistics, the data points behind means, medians and variance measures should be available. If there are restrictions on publicly sharing data—e.g. participant privacy or use of data from a third party—those must be specified.

 Reviewer #1: Yes

Reviewer #2: Yes

 4. Is the manuscript presented in an intelligible fashion and written in standard English?

PLOS ONE does not copyedit accepted manuscripts, so the language in submitted articles must be clear, correct, and unambiguous. Any typographical or grammatical errors should be corrected at revision, so please note any specific errors here.

 Reviewer #1: Yes

Reviewer #2: Yes

 5. Review Comments to the Author

Please use the space provided to explain your answers to the questions above. You may also include additional comments for the author, including concerns about dual publication, research ethics, or publication ethics. (Please upload your review as an attachment if it exceeds 20,000 characters)

Reviewer #1: I am thrilled to see a manuscript with this subject being considered for publication. Trauma-informed care (TIC) is a well-established framework for providing quality clinical care to survivors of various forms of trauma. TIC is rapidly growing in popularity and will soon affect the practice of all subspecialties of medicine. The authors have done an excellent job in conceptualizing and studying trauma-informed interventions that are proposed to impact patient care. The main strength of this article is its inclusion of perspectives from both physicians and patients. The data noted in the attached Tables were helpful and interesting. I have included comments throughout the manuscript with suggestions that I hope will be useful to the authors. One recommendation that I have is to include all introductory information re: trauma, TIC, definitions and epidemiology early on in the manuscript. It would also help to clarify how the authors selected their own key principles of TIC that differ slightly from SAMHSA's. I have also made recommendations for the authors to consider noting other published work on this subject that may be helpful and/or related. References to concepts such as emotional intelligence training in the manuscript can be more specifically tied to TIC (i.e. see Sanctuary Model, 2008). There were several instances throughout the discussion sections where I felt that the voice / insight / explanations of the primary care physician was not as clear as I would have hoped. I wonder whether collaboration with a PCP might be helpful in strengthening the interpretation of the results in the manuscript. Overall, this piece makes an important contribution to the field and would be wonderful to publish following edits as suggested. Thank you for the opportunity to review this paper.

*We thank this reviewer for their positive response to our manuscript. The comments provided throughout the manuscript were helpful and we have addressed the reviewer’s suggestions. As suggested, we me included introductory information earlier on in the manuscript and clarified how our key principles of TIC aligned with SAMHSA’s. We reviewed references that were suggested and incorporated them into the discussion as appropriate. We hope that the revised discussion sections are clearer.*

Reviewer #2: Very well written and sound methodologies. The only question I have is regarding the measurement instrument, is it validated. If not, why the research team decided to use this approach--should also note as a limitation if not validated.

*We thank this reviewer for their feedback. We have now noted that the measurement instrument has not been validated and as such is a limitation. In the Strengths and limitations section, we also added the reason we decided to use this approach.*

 6. PLOS authors have the option to publish the peer review history of their article ([what does this mean?](https://journals.plos.org/plosone/s/editorial-and-peer-review-process#loc-peer-review-history)). If published, this will include your full peer review and any attached files.


If you choose “no”, your identity will remain anonymous but your review may still be made public.


**Do you want your identity to be public for this peer review?** For information about this choice, including consent withdrawal, please see our [Privacy Policy](https://www.plos.org/privacy-policy).

 Reviewer #1: No

Reviewer #2: No
