# Supplementary material for: A patient-oriented research approach to assessing patients’ and primary care physicians’ opinions on trauma-informed care
Source: PLoS One. 2021 Jul 9;16(7):e0254266. doi: 10.1371/journal.pone.0254266 (PMC8270182; doi:10.1371/journal.pone.0254266)
Supplement: Attachment — Submitted filename: Response to Reviewers .docx [file pone.0254266.s006.docx]

Reviewer #1: -I very much appreciate the authors’ hard work in revising this manuscript. It was a completely different experience reading this version compared to the first. Impressive revision indeed! I would be likely to recommend this article to peers in TIC.

-The introduction is much clearer and helpful in defining TIC. The only area for improvement in the Intro is the final paragraph of the introduction. I would define Phase 1 with slightly more detail (ex. “In Phase 1, the research team examined physician and patient opinions of TIC” as well as Phase 2 “…for Phase 2’s examination of physician and patient recommendations for TIC applications”). Otherwise, references to Phase 1 and 2 in the intro lack meaning and leave lingering questions for the reader. Also in this paragraph, “the framework and six core principles” should be further clarified—whose framework? And SAMHSA’s six core principles? It would be best to specify “paraphrased by our team as…”. I would also expand the relevance of each of your principles from “patients” to include everyone in the system, because in a true trauma-informed organization (as defined in Tip 57), SAMHSA describes the principles as relevant for application to clients, family, staff, etc. For Cultural Sensitivity, consider adding a word that goes beyond awareness of biases and includes addressing them.

-The Phase 1 Methods are very well described.

-I love the focus on the IMPACT of trauma in the Discussion section of Phase 1. Physicians may have rated inquiry low b/c of unfamiliarity with trauma screening (PCPs in the U.S. are not trained in this at all)—might be helpful to note that. The sentence “Whereas simply knowing…” doesn’t seem like a complete sentence. There’s a key opportunity in this paragraph to mention “universal precautions” with TIC—it may not actually be relevant to know your pt’s trauma hx, if you apply these principles universally.

-In the Phase 2 section, 1st paragraph, would clarify “…patients did not perceive the __importance of?__ receiving TIC at the same level…”

-In the Phase 2 Results 3rd paragraph, would add an apostrophe for “…lower than physicians’…”

-In the final paragraph of the Phase 2 Results section, it would make more sense to switch these 2 sentences, as the phrase “clinical pathway” comes as a surprise to readers (hasn’t yet been mentioned). It would also be helpful to define what you mean by clinical pathway and what role it played in your study.

-In the Conclusion of the paper, I might adjust the 3rd sentence to more accurate reflect your results. Consider something to the effect of “…as pts find TIC important but do not perceive receiving it frequently.” I would strongly consider ending the conclusion on a positive note, rather than closing with a statement that includes the phrase “cost-effective”. Why do you think TIC is important? Do you think it’s possible to implement these strategies you’ve identified? What do you think would be the ultimate impact on physicians and patients, or healthcare on the whole?

*We thank this review for all their helpful and supportive feedback. We addressed each of the comments in the respective sections of the manuscript and provided a version with track changes to highlight the revisions made. As suggested, we included a statement about paraphrasing SAMHSA’s TIC principles, we delineated the role of clinical pathways, and we expanded on the conclusion. We hope this revised draft is clearer.*

Reviewer #3: This is an important investigation - there must be an understanding of how both the patient and the provider view TIC in order to increase the probability that the two will connect in a way that enhances the healing experience of the patient and gives instruction to the provider regarding how better to provide care. This study shows areas of concordance and discord between the two groups, which is a good starting point. The discussion of the reasons behind the answers endorsed be each group is necessarily speculative 9in the absence of focus groups or another method to get to the thought processes of providers and patients leading to the results. The discussion suggests that social determinants of health and historical institutionalized racism can be viewed through a trauma lens and this is likely a fruitful area for future work.

*We thank this reviewer for their supportive feedback. Future research will be needed to investigate how the recommendations proposed will improve patient care.*
